# Human dental pulp pluripotent-like stem cells promote wound healing and muscle regeneration

**DOI:** 10.1186/s13287-017-0621-3

**Published:** 2017-07-27

**Authors:** Ester Martínez-Sarrà, Sheyla Montori, Carlos Gil-Recio, Raquel Núñez-Toldrà, Domiziana Costamagna, Alessio Rotini, Maher Atari, Aernout Luttun, Maurilio Sampaolesi

**Affiliations:** 1 Regenerative Medicine Research Institute, Universitat Internacional de Catalunya, Barcelona, 08017 Spain; 2 Translational Cardiomyology Laboratory, Stem Cell Biology and Embryology Unit, Department of Development and Regeneration, KU Leuven, Leuven, 3000 Belgium; 3 Department of Neuroscience, Imaging and Clinical Sciences, University “G. d’Annunzio”, Chieti, 66100 Italy; 4 Interuniversity Institute of Myology, Chieti, 66100 Italy; 5 Centre for Molecular and Vascular Biology, Department of Cardiovascular Sciences, KU Leuven, Leuven, 3000 Belgium; 6 Human Anatomy Unit, Department of Public Health, Experimental and Forensic Medicine, University of Pavia, Pavia, 27100 Italy

**Keywords:** Dental pulp, Stem cells, Revascularisation, Angiogenesis, Wound healing, Muscular dystrophy, Growth factors, Cytokines

## Abstract

**Background:**

Dental pulp represents an easily accessible autologous source of adult stem cells. A subset of these cells, named dental pulp pluripotent-like stem cells (DPPSC), shows high plasticity and can undergo multiple population doublings, making DPPSC an appealing tool for tissue repair or maintenance.

**Methods:**

DPPSC were harvested from the dental pulp of third molars extracted from young patients. Growth factors released by DPPSC were analysed using antibody arrays. Cells were cultured in specific differentiation media and their endothelial, smooth and skeletal muscle differentiation potential was evaluated. The therapeutic potential of DPPSC was tested in a wound healing mouse model and in two genetic mouse models of muscular dystrophy (*Scid/mdx* and *Sgcb-null Rag2-null γc-null*).

**Results:**

DPPSC secreted several growth factors involved in angiogenesis and extracellular matrix deposition and improved vascularisation in all three murine models. Moreover, DPPSC stimulated re-epithelialisation and ameliorated collagen deposition and organisation in healing wounds. In dystrophic mice, DPPSC engrafted in the skeletal muscle of both dystrophic murine models and showed integration in muscular fibres and vessels. In addition, DPPSC treatment resulted in reduced fibrosis and collagen content, larger cross-sectional area of type II fast-glycolytic fibres and infiltration of higher numbers of proangiogenic CD206^+^ macrophages.

**Conclusions:**

Overall, DPPSC represent a potential source of stem cells to enhance the wound healing process and slow down dystrophic muscle degeneration.

**Electronic supplementary material:**

The online version of this article (doi:10.1186/s13287-017-0621-3) contains supplementary material, which is available to authorized users.

## Background

Dental pulp pluripotent-like stem cells (DPPSC) [1] have been recently isolated from the dental pulp of human third molars and express pluripotency markers such as OCT3/4, SOX2 and NANOG. DPPSC show pluripotent-like behaviour, as they can differentiate in vitro into tissues of the three embryonic germ layers and are able to form embryoid body-like and teratoma-like structures [2]. This population represents an easily accessible autologous source of adult stem cells and is not touched by the ethical controversy that is associated with the use of embryonic stem cells.

The restoration of organ function requires effective revascularisation, the generation of new blood vessels formed mainly by endothelial cells (ECs) and smooth muscle cells (SMCs). Currently, there is a growing list of highly prevalent diseases with a blood vessel-related aetiology [3]. Indeed, more than 500 million people worldwide would benefit from pro- or anti-angiogenic treatments [4]. Moreover, blood vessels represent an essential component of tissue engineering. If blood supply cannot be rapidly established in an implanted tissue, necrosis will occur due to insufficient oxygen and nutrient transport [5]. So far, available vascular grafts cannot reproduce the complex functions of native vessels [6], and in this regard it has been proven that the presence of functional ECs in the grafts can improve these functions [7]. Since autologous sources of ECs are insufficient or very difficult to obtain [8], stem cell differentiation has been explored in order to increase their number [9–12]. Tissue regeneration and stimulation of blood vessel growth are critical factors for the successful treatment of wounds [13] and skeletal muscle degeneration [14]. Wound healing is a complex and organised process that restores integrity and function of the damaged tissue [15]. It restores the barrier function of the skin by re-epithelialisation, revascularisation, matrix deposition/remodelling and contraction. These processes involve the interaction of different cell types including ECs, SMCs, platelets, inflammatory cells, (myo)fibroblasts and keratinocytes. These cells secrete vascular endothelial growth factor (VEGF), basic fibroblast growth factor (bFGF), platelet-derived growth factor (PDGF), placental growth factor (PlGF), transforming growth factor beta (TGF-β) and tissue inhibitor of metalloproteinases 2 (TIMP-2) among other factors to regulate the healing process and modulate the extracellular matrix components [16–18]. For healing of large wound defects, autologous tissue-engineered skin grafts are needed. After grafting, quick and functional vascularisation needs to be achieved to help tissue-engineered skin to survive [19]. The use of mesenchymal stromal cells (MSCs) has been shown to promote wound healing through different processes, including angiogenesis [20]. Muscular dystrophies (MDs) are genetic diseases that cause progressive weakness and degeneration of skeletal muscles. Mutations in the dystrophin gene underlie Duchenne muscular dystrophy (DMD), the most common and severe form of muscular dystrophies. Other dystrophies feature mutations in genes encoding sarcoglycans [21] and are included in the group of limb-girdle muscular dystrophies (LGMDs). LGMD type 2E (LGMD2E), with a mutation in the beta-sarcoglycan (*SGCB*) gene, represents a severe form of LGMD [22, 23], often associated with cardiomyopathy and abnormalities in the vasculature [23–25]. Currently, there is no cure for DMD or LGMD2E [26, 27] and effective therapeutic approaches capable of counteracting muscle and vessel degeneration are needed. In this regard, stem cell therapy can be used to restore dystrophin or beta-sarcoglycan in dystrophic muscles, thereby repairing the damaged fibres and preventing future muscle degeneration.

In this study, we investigated the therapeutic potential of DPPSC in a model of wound healing in nude mice, and in immunodeficient mice resembling DMD and LGMD2E disorders, *Scid/mdx* [28] and *Sgcb-null Rag2-null γc-null* [29], respectively.

## Methods

### Patient selection

DPPSC were isolated from healthy human third molars extracted for orthodontic and prophylactic reasons from 15 patients with ages between 14 and 21 years old. All patients (or their legal guardians) provided informed consent before obtaining the samples. This study was approved by the Committee on Ethics in Research (CER) of the Universitat Internacional de Catalunya (Spain) under the protocol code BIO-ELB-2013-04.

### Isolation and culture of DPPSC

DPPSC were extracted and isolated as previously described [2]. Briefly, teeth were washed after extraction using gauze soaked in 70% ethanol and dental pulp was extracted from the teeth using a sterile nerve-puller file 15 and forceps (if the apexes were still open) or fracturing the teeth and taking the dental pulp using forceps. The dental pulp was placed in sterile 1X phosphate-buffered saline (PBS; Life Technologies, Carlsbad, CA, USA) with 5% of 0.25% trypsin-EDTA (Life Technologies) and 1% penicillin-streptomycin (Life Technologies) and transferred to the laboratory. The tissues were disaggregated by digestion with collagenase type I (3 mg/mL; Sigma-Aldrich, St. Louis, MO, USA) for 60 minutes at 37 °C. Obtained cells were cultivated in DPPSC medium, which consisted of 60% Dulbecco’s modified Eagle’s medium (DMEM)-low glucose (Life Technologies) and 40% MCDB-201 (Sigma-Aldrich) supplemented with 1 × insulin-transferrin-selenium (ITS; Sigma-Aldrich), 1 × linoleic acid-bovine serum albumin (LA-BSA; Sigma-Aldrich), 10^-9^ M dexamethasone (Sigma-Aldrich), 10^-4^ M ascorbic acid 2-phosphate (Sigma-Aldrich), 100 units of penicillin/1000 units of streptomycin (Life Technologies), 2% foetal bovine serum (FBS; Sigma-Aldrich), 10 ng/mL human PDGF-BB (Abcam, Cambridge, UK), 10 ng/mL epidermal growth factor (EGF; R&D Systems, Minneapolis, MN, USA), 1000 units/mL human leukemia inhibitory factor (LIF; EMD Millipore, Billerica, MA, USA), Chemically Defined Lipid Concentrate (Life Technologies), 0.8 mg/mL BSA (Sigma-Aldrich) and 55 mM β-mercaptoethanol (Sigma-Aldrich) in 650 mL flasks precoated overnight with 100 ng/mL fibronectin at 37 °C in a 5% CO_2_ incubator. During the 2 weeks of primary culture, the medium was changed every 4 days. To propagate DPPSC, the cells were detached at 30% confluence by adding PBS containing 0.25% trypsin-EDTA (Life Technologies) and replated at a density of 100–150 cells/cm^2^. Cell populations from different donors at passage 5 and passage 10 were negative for mycoplasma contamination (MycoAlert™ Mycoplasma Detection Kit, Lonza, Basel, Switzerland).

### Culture of HUVECs

Human umbilical vein endothelial cells (HUVECs; Lonza; tested for mycoplasma, bacteria, yeast, fungi, HIV-1, hepatitis B and hepatitis C) were maintained in Endothelial Growth Medium 2 (EGM-2; Lonza). The medium was changed every 2 days and the cells were passaged when they reached 70–85% confluence using PBS containing 0.25% trypsin-EDTA (Life Technologies) and replated at a density of 2.5 × 10^3^ cells/cm^2^.

### Culture of C2C12 cells

The mouse immortalised myoblast cell line C2C12 was maintained using DMEM 4.5 g/L glucose supplemented with 10% FBS (Hyclone, South Logan, UT, USA), 1% glutamine (Sigma-Aldrich), 1% sodium pyruvate (Life Technologies) and 1% penicillin/streptomycin (Life Technologies). Cells were negative for mycoplasma contamination (MycoAlert™ Mycoplasma Detection Kit, Lonza).

### Lentiviral transduction

DPPSC were seeded at a density of 500 cells/cm^2^ and transduced at 60% confluence with lentiviruses encoding for turbo green fluorescent protein (tGFP) from Pontellina Plumata for 24 hours. Briefly, incompetent viral particles were produced in packaging cells (HEK293T) by co-transfecting the tGFP lentiviral vector plasmid (Sigma-Aldrich shc003) with compatible Lentiviral Packaging Mix (Sigma-Aldrich). This method allowed generation of 100% GFP^+^ DPPSC that were used for the wound healing assay.

### In vitro EC differentiation

DPPSC were seeded in 24-well plates at 2 × 10^4^ cells/cm^2^ and differentiated using EGM-2 (Lonza) conditioned for 24 h with HUVECs (and then filtrated with 0.22-μm diameter pores to eliminate any HUVECs). The medium was changed every 2–3 days for 28 days. RNA extraction and Matrigel™ assay were performed at day 7, 14, 21 and 28 of differentiation. Immunofluorescence analysis was performed at day 28. For the Matrigel™ assay, DPPSC were detached using PBS containing 0.25% trypsin-EDTA (Life Technologies) and 50,000 cells were replated in EGM-2 medium in a 24-well coated with Matrigel™ (Basement Membrane Matrix Growth Factor Reduced; BD Biosciences, San Jose, CA, USA). Matrigel™ coating was performed following manufacturer’s instructions for the Thin Gel Method assay, adding 250 μl of Matrigel™ in a 24-well and keeping it 30 minutes at 37 °C. After 24 hours, tube-like structures were analysed and pictures were taken with an OX.3040 Euromex binocular microscope for phase contrast using the camera DC.10000c CMEX-10 digital 10 Mpix USB-2 CMOS (Euromex, Arnhem, The Netherlands).

### In vitro SMC differentiation

DPPSC from two different donors and two passages (passage 5 and passage 10) were differentiated to SMCs using differentiation media consisting of High-Glucose DMEM (Life Technologies) supplemented with 2% horse serum (Life Technologies), 1% sodium pyruvate (Life Technologies), 1% glutamine (Life Technologies), 1% penicillin-streptomycin (Life Technologies) and 50 ng/mL TGF-β1 (Peprotech, Rocky Hill, NJ, USA). Cells were plated in 24-well plates at 500 cells/cm^2^ and differentiation was started when they reached 60% confluence. Medium was changed every 2–3 days for 10 days. tGFP^+^ DPPSC from one of the donors were also differentiated using the same conditions.

### In vitro skeletal muscle differentiation

DPPSC from three different donors and two passages (passage 5 and passage 10) were differentiated to skeletal muscle using differentiation media consisting of High-Glucose DMEM (Life Technologies) supplemented with 2% horse serum (Life Technologies), 1% sodium pyruvate (Life Technologies), 1% glutamine (Life Technologies) and 1% penicillin-streptomycin (Life Technologies). Cells were differentiated alone or in direct co-culture with C2C12 cells at 1:1 ratio. The cells were plated in 24-well plates at a density of 1 × 10^4^ cells/cm^2^ and induction was started when cells reached 70% confluence. Medium was changed every 2–3 days for 5–7 days. tGFP^+^ DPPSC from one of the donors were also differentiated in the same conditions. Fusion index was calculated as the ratio of the number of nuclei inside the MyHC^+^ myotubes to the number of total nuclei (percentage).

### Wound healing assay

Eight-week-old male athymic nude mice (*Foxn1*, Charles River Laboratories, Wilmington, MA, USA) were treated with DPPSC (P10) or PBS (*n* = 5 for each condition). The mice were injected with anti-NK to eliminate natural killer cell activity 24 hours before starting the surgery. Full-thickness wounds (0.5-cm diameter) were made on the back of the mice, splinted with silicone rings and treated with tGFP^+^ DPPSC (1 × 10^6^ cells per mouse) or PBS. A Tegaderm™ dressing (3M, Maplewood, MN, USA) was applied to prevent the wound area from drying out. All wounded mice were housed individually to avoid fighting and to prevent removal of the occlusive wound dressing. Every other day, digital pictures of the wounds were taken (using a NikonD1 camera and Camera-Control-Pro software; Nikon, Tokyo, Japan) under isoflurane anaesthesia and the dressings were renewed. Presence of DPPSC was monitored at day 5 and 10 using a Zeiss SteREO Discovery.V12 microscope and Axio Imaging software (Carl Zeiss, Oberkocken, Germany). Wound contraction was evaluated by comparing relative wound area (RWA) over time. RWA was calculated using ImageJ software (NIH, Baltimore, MD, USA) by dividing the healing wound area by the fixed reference area inside the silicone ring and expressing it as a percentage. To account for small inter-animal variations, for each time point, relative wound area of each individual animal was expressed as percentage compared to the relative wound area at day 0. Wound contraction (%) was calculated as the complement of relative wound area (100-RWA). At day 11 after wounding, mice were sacrificed and skin fragments including the wound area and a rim of normal skin were dissected out, fixed, separated in two pieces at the midline and processed for paraffin embedding. For all stainings and analyses, 7 μm microtome sections were used. Mouse procedures were approved by the ethics committee for use of animals in research from KU Leuven (Belgium) under the protocol code ECD N°P018/2015.

### DPPSC injection in dystrophic mice

Ten 3-month-old *Scid/mdx* mice, five males and five females, and four 3-month-old *Sgcb-null Rag2-null γc-null* mice, three males and one female, were injected in the left tibialis anterior with 2.5 × 10^5^ cells per mouse. DPPSC from P10 were used. Untreated right limbs were used as controls. After 20 days, mice were sacrificed and muscles were frozen and kept at -80 °C. The samples were then cut in 7 μm sections using a cryostat (Leica, Wetzlar, Germany). Mouse procedures were approved by the ethics committee for use of animals in research from KU Leuven (Belgium) under the protocol code ECD N°P095/2012.

### Short-comparative genomic hybridisation

The short-comparative genomic hybridisation (sCGH) technique was performed as described in Rius et al. [30] catching single cells from a homogeneous DPPSC culture. All samples were analysed in triplicate. The DNA control used for the hybridisation was XXY.

### Quantitative reverse transcription (qRT)-PCR analysis

Samples of total RNA were extracted using Trizol (Life Technologies) and isolated following manufacturer’s instructions. Two μg of total RNA with a ratio 260/280 between 1.8 and 2 were treated with DNase I (Life Technologies) and reverse-transcribed using Transcriptor First Strand cDNA Synthesis Kit (Roche, Basel, Switzerland). Polymerase chain reaction (PCR) was performed using the primers in Additional file 1: Table S1 for the amplification of the desired cDNA using FastStart Universal SYBR Green Master (Roche) for Real-Time PCR using a CFX96 Real-Time PCR Detection System (Bio-Rad, Hercules, CA, USA). The expression levels of the genes of interest were normalised against the housekeeping gene *GAPDH* and the relative expression levels were then normalised to undifferentiated DPPSC at P5 or at day 0 of differentiation, which were assigned as 1. All analyses were performed using the 2^(-ΔΔCT)^ method and three technical replicates.

### Cytokine antibody arrays

Undifferentiated DPPSC (1.5 × 10^6^ cells) from three different donors were cultured for 48 hours in DPPSC medium without FBS, LIF or BSA. tGFP^+^ DPPSC were also used. The presence of several growth factors and cytokines secreted in the medium was analysed using human cytokine antibody arrays containing 80 target proteins (Abcam ab133998). Briefly, the supplied membranes (spotted with 80 different antibodies) were blocked with the provided buffer and subsequently incubated at 4 °C overnight with 1 ml of the DPPSC medium in contact with DPPSC for 48 hours. One of the membranes was incubated with an uncultured medium and used as a control. Membranes were washed with the provided buffers and incubated again at 4 °C overnight with the supplied biotin-conjugated anti-cytokines. The arrays were then washed, incubated with horseradish-peroxidase-conjugated streptavidin for 2 hours at room temperature and washed. Chemiluminescence reaction was detected using the supplied detection buffers. Semi-quantification by relative densitometry was obtained using Quantity One software (Bio-Rad) and normalised to the positive control signals in each membrane for comparison of multiple arrays and to EGF.

The presence of cytokines in lysates from DPPSC-injected and control muscles of dystrophic mice (three *Sgcb-null Rag2-null γc-null* mice and one *Scid/mdx* mouse) was also analysed using mouse cytokine antibody arrays containing 22 target proteins (Abcam ab133993). Briefly, muscle lysates were obtained by mechanical homogenisation in RIPA buffer (Sigma-Aldrich) supplemented with and 1 mM phenylmethanesulfonyl fluoride, 0.5 mM sodium orthovanadate, 1:100 protease inhibitor cocktail and 10 mM sodium fluoride (Sigma-Aldrich) using a T10 basic Ultra-Turrax homogeniser (IKA, Staufen, Germany). Subsequently, samples were sonicated (Branson digital sonifier 250; Branson Ultrasonics, Danbury, CT, USA) two times for 10 seconds each, kept on ice for 30 minutes, centrifuged at 13000 × g for 10 minutes at 4 °C and supernatants were collected. Protein concentrations were determined using the Bradford assay (Bio-Rad) and equal amounts of protein (300 μg) were incubated at 4 °C overnight with the supplied membranes (previously blocked). Subsequent steps were performed following the same protocol used with the human cytokine arrays.

### Immunofluorescence analyses

Cryosections and cells were fixed with 4% paraformaldehyde for 15 minutes or ethanol-acetone 1:1 for 4 minutes at room temperature and then, after three PBS washes, incubated with 1% BSA + 0.2% or 0.5% triton for 30–45 minutes at room temperature to increase permeability. Cells were then incubated for 30–60 minutes with donkey serum at room temperature and, after that, 2 hours at room temperature or 4 °C overnight with the primary antibody (Additional file 2: Table S2). The following day, after three PBS washes, they were incubated for 1–2 hours at room temperature with the secondary antibody and washed again three times. DAPI (1:3000) was used for nuclear counterstaining, washed three times and wells containing cells or slides were mounted using FluorSave™ mounting medium (EMD Millipore). Paraffin-embedded sections were deparaffinised and rehydrated, followed by an antigen recovery step (putting the slides in citrate buffer pH = 6 for 20 minutes in the microwave, in trypsin 1:80 in 0.01% CaCl_2_ at 37 °C for 7 minutes or in target retrieval solution (Dako, Glostrup, Denmark) for 20 minutes at 95 °C). Samples were washed with Tris-buffered saline (TBS), incubated for 20 minutes with MeOH-H_2_O_2_ and washed again. They were then permeabilised using 0.5% triton, washed with TBS, blocked in 20% donkey serum or Tris-NaCl blocking buffer and incubated with the primary antibody (Additional file 2: Table S2) 2 hours or overnight at room temperature. Samples were washed with Tris-NaCl-Tween buffer, incubated with the secondary antibody and washed again. CD31, laminin and collagen type I and III signals were amplified using TSA Fluorescein System (Perkin Elmer, Waltham, MA, USA). Samples were mounted using Prolong Gold with DAPI (Life Technologies). Pictures were taken with an Eclipse Ti Microscope (Nikon) and NIS-Elements AR 4.11 software. Images were merged and/or quantified using ImageJ software (NIH).

### Alkaline phosphatase staining

SIGMAFAST™ BCIP®/NBT (Sigma-Aldrich) was used following manufacturer’s instructions. Briefly, one tablet was dissolved in 10 mL of water, and 1 mL was added in one 24-well plate containing undifferentiated DPPSC. The solution was kept for 2 hours at 37 °C and the wells were washed afterwards with PBS. Fibroblasts were used as a negative control.

### Haematoxylin and eosin staining

Paraffin sections were heated at 57 °C for 60 minutes, deparaffinised and rehydrated. Cryosections were fixed with 4% paraformaldehyde for 15 minutes. The samples were then soaked in distilled water for 5 minutes, Harris haematoxylin for 4 minutes and washed afterwards in running tap water for 2 minutes. The sections were subsequently soaked for 1 minute each in acid alcohol, running water, bluing reagent, running water, eosin, 95% ethanol, 100% ethanol and histoclear. The slides were then mounted with DPX and left on a slide heater overnight. Pictures from paraffin sections were taken with a Leica DMRBE microscope connected with an AxioCam MRc5 camera (Zeiss). Pictures from cryosections were taken with a Nikon Eclipse Ti microscope (Nikon). Epithelial coverage and thickness and cross-sectional area were determined using ImageJ software (NIH).

### Sirius red staining

Sirius Red solution was prepared by mixing 0.2 g of Direct Red 80 (Sigma-Aldrich) with saturated aqueous solution of picric acid (prepared mixing 8 g of picric acid in 200 mL of distilled water). Paraffin sections were deparaffinised and rehydrated and crysections were fixed with 4% paraformaldehyde for 15 minutes. Sections were put in tap water for 10 minutes, distilled water for 5 minutes and Sirius Red solution for 90 minutes. After that, the slides were washed with HCL 0.01 N for 2 minutes and dehydrated with ethanol 70% for 45 seconds and ethanol 100% for 5 minutes (twice). Lastly, samples were cleared in xylol for 5 minutes (twice), mounted with DPX and left on a slide heater overnight. Pictures were taken with a Leica DMRBE microscope connected with an AxioCam MRc5 camera (Zeiss). Collagen quantification was determined using ImageJ software (NIH).

### Masson’s trichrome staining

Cryosections were fixed with 4% paraformaldehyde for 15 minutes and subsequently incubated for 15 min at 57 °C in Bouin solution. Afterwards, cryosections were stained in Working Weigert's Iron Hematoxylin Solution for 5 minutes, washed in running tap water for 5 minutes and stained in Biebrich Scarlet-Acid Fucsin for 5 minutes. Slides were subsequently soaked in de-ionised water and in Working Phosphotungstic\Phosphomolybdic acid solution for 5 minutes and stained in Aniline Blue solution for 5 minutes and acid acetic 1% for 2 minutes. Slides were mounted and left in a slide heater overnight. Pictures were taken on a Nikon Eclipse Ti microscope (Nikon). Fibrotic area was quantified using ImageJ software (NIH).

### Esterase staining

Cryosections were rehydrated and left in PBS for 5 minutes. A staining solution of 5 mL containing 2 mg NADH (Sigma-Aldrich) with 4 mg NBT (Roche) in 4.5 mL H_2_O and 0.5 mL 1 M Tris-HCl pH = 7.5 was prepared. Drops of prepared solution were added to the sections, which were then incubated for 30–60 minutes at 37 °C incubator. Samples were washed with H_2_O twice, dehydrated in ascending scale of alcohol (50%, 80%, 90% and 100%) for 1 minute each and then alcohol was removed with histoclear. Slides were mounted with DPX and left to dry in a heater. Pictures were taken on a Nikon Eclipse Ti microscope (Nikon). Cross-sectional area was calculated using ImageJ software (NIH).

### Statistical analyses

Data from the different experiments were analysed using the statistical program Statgraphics Centurion XVI (Statgraphics, Warrenton, VA, USA). Two-tailed *t* test or one-way ANOVA were used to compare interrelated samples, while two-way ANOVA was used to analyse multiple factors. Confidence intervals were fixed at 95% (*p* < 0.05), 99% (*p* < 0.01) and 99.9% (*p* < 0.001). GraphPad Prism (GraphPad Software, San Diego, CA, USA) was used to graph the results as the mean ± standard error of the mean (s.e.m.). Refer to figure legends for specific information regarding the statistical test used and the number of independent experiments or biological replicates.

## Results

### Characterisation of DPPSC and their in vitro differentiation potential

**Fig. 1:**
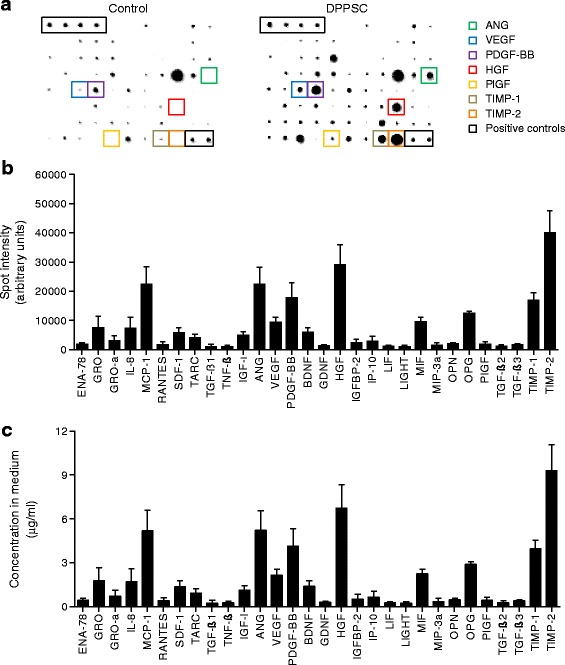
Growth factor and cytokine secretion by DPPSC. **a** Representative images of cytokine antibody arrays showing the presence of several growth factors in DPPSC-conditioned medium (*right panel*) compared to uncultured media (*left panel*). **b** Analysis of the increase in spot intensity (in arbitrary units; AU) in DPPSC-cultured vs uncultured media. Only increments higher than 1000 AU are shown in the figure (*n* = 4, results are displayed as mean ± s.e.m.). **c** Concentration (μg/ml) of proteins in **b** in the DPPSC-cultured medium (*n* = 4, results are displayed as mean ± s.e.m.). *Abbreviations*: *ENA-78* C-X-C motif chemokine 5 (CXCL5), *GRO* growth-regulated oncogene, *GRO-α* growth-regulated oncogene alpha, *IL-8* interleukin 8, *MCP-1* monocyte chemoattractant protein 1, *RANTES* regulated on activation normal T cell expressed and secreted (CCL5), *SDF-1* stromal cell-derived factor 1, *TARC* thymus and activation regulated chemokine, *TGF-β1* transforming growth factor beta 1, *TNF-β* tumor necrosis factor beta, *EGF* epidermal growth factor, *IGF-I* insulin-like growth factor 1, *ANG* angiogenin, *VEGF* vascular endothelial growth factor, *PDGF-BB* platelet-derived growth factor-BB, *BDNF* brain-derived neurotrophic factor, *GDNF* glial cell-derived neurotrophic factor, *HGF* hepatocyte growth factor, *IGFBP-2* insulin-like growth factor-binding protein 2, *IP-10* interferon gamma-induced protein 10, *LIF* leukemia inhibitory factor, *LIGHT* TNF superfamily member 14 (TNFSF14), *MIF* macrophage migration inhibitory factor, *MIP-3α* macrophage inflammatory protein-3, *OPN* osteopontin, *OPG* osteoprotegerin, *PlGF* placental growth factor, *TGF-β2* transforming growth factor beta 2, *TGF-β3* transforming growth factor beta 3, *TIMP-1* tissue inhibitor of metalloproteinases 1, *TIMP-2* tissue inhibitor of metalloproteinases 2

As described before, DPPSC had a small size with large nuclei and low cytoplasm content and this morphology was maintained for more than 15 passages (Additional file 3: Figure S1a-d). The growth rate of DPPSC populations from 13 different donors until passage 15 was analysed (Additional file 3: Figure S1e, f). Ten DPPSC populations were analysed by short-comparative genomic hybridisation (sCGH) and none of them showed chromosomal abnormalities (a representative example is shown in Additional file 3: Figure S1g). DPPSC expressed pluripotency markers *OCT4A* and *NANOG* in all passages analysed, although their expression was found to be significantly higher at passage 10 compared to other passages (Additional file 3: Figure S1h). Immunofluorescence analyses confirmed the presence of NANOG and SOX2 proteins in DPPSC (Additional file 3: Figure S1i, j). DPPSC were also positive for alkaline phosphatase (ALP) enzymatic staining (Additional file 3: Figure S1k). To get insights into DPPSC secretion we analysed which molecules can be produced by these cells. Analysis with human cytokine antibody arrays showed angiogenin (ANG), VEGF, PDGF-BB, hepatocyte growth factor (HGF), monocyte chemoattractant protein 1 (MCP-1), osteoprotegerin, tissue inhibitor of metalloproteinases 1 (TIMP-1) and TIMP-2 as main molecules secreted by DPPSC (Fig. 1a, b). The quantification of these molecules revealed that DPPSC released in the medium more than 4 μg/ml of ANG, PDGF-BB, HGF, MCP-1, TIMP-1 and TIMP-2 (Fig. 1c).

**Fig. 2:**
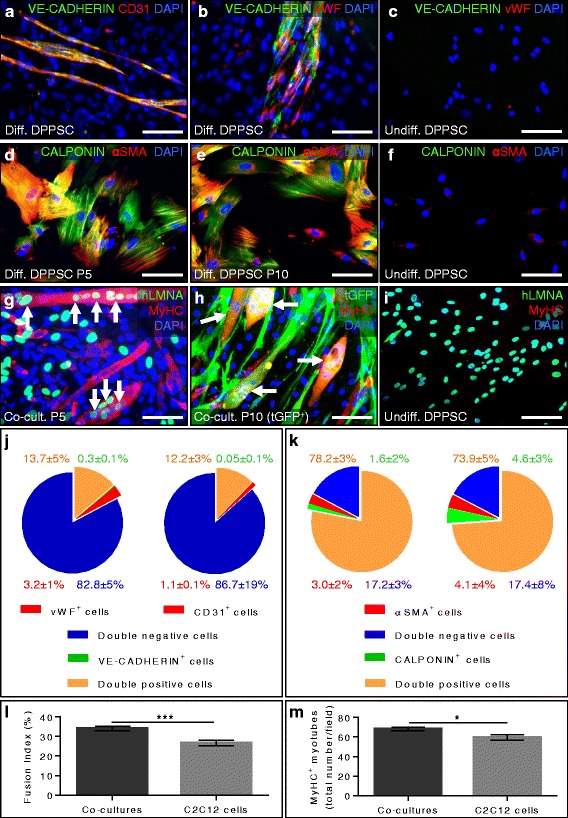
In vitro mesodermal differentiation of DPPSC. **a**-**c** Immunofluorescence analysis for VE-cadherin (*green*; **a**, **b**), CD31 (*red*; **a**) and vWF (*red*; **b**) in DPPSC differentiated for 28 days to the endothelial lineage (**a**, **b**); undifferentiated DPPSC were used as negative control (**c**). **d**-**f** Immunofluorescence analysis for the smooth muscle markers calponin (*green*) and αSMA (*red*) in differentiated DPPSC from passage (P) 5 (**d**) and P10 (**e**) cultured in smooth muscle differentiation medium for 10 days; undifferentiated DPPSC were used as negative control (**f**). **g** Immunofluorescence analysis of DPPSC co-cultured with C2C12 cells at day 5 of myogenic induction. *Arrows* indicate the presence of human nuclei (stained for hLMNA in *green*), inside the formed myotubes expressing MyHC (*red*). **h** Immunofluorescence analysis of tGFP^+^ DPPSC (*green*) co-cultured with C2C12 cells. *Arrows* indicate MyHC^+^ chimeric myotubes. **i** Immunofluorescence analysis for hLMNA (*green*) and MyHC (*red*) in undifferentiated DPPSC was used as a negative control. For **a**-**i**, nuclei are counterstained with DAPI (*blue*). Scale bars: 100 μm. **j** Quantitative analysis of DPPSC differentiated towards the endothelial lineage expressing VE-cadherin (*green*), vWF or CD31 alone (*red*), double positive for VE-cadherin and CD31 (*left graph in orange*) or for VE-cadherin and vWF *(right graph in orange*), or none of these markers (*blue*; *n* = 3 independent experiments). **k** Quantitative analysis of DPPSC differentiated towards the smooth muscle lineage expressing calponin (*green*), αSMA (*red*), both smooth muscle markers (*orange*) or none of these markers (*blue*) at P5 (*left graph*) and P10 (*right graph*). No statistically significant difference was found (*n* = 3 independent experiments). **l** Fusion index analysis in DPPSC-C2C12 co-cultures (***p < 0.001, *n* = 6 independent experiments). **m** Quantitative analysis of the number of MyHC^+^ myotubes in DPPSC-C2C12 co-cultures (*p < 0.05, *n* = 6 independent experiments). For **k**-**m**, two-tailed Student’s *t* test was used and results are displayed as mean ± s.e.m

DPPSC subjected to culture conditions favouring endothelial differentiation showed a significant up-regulation of the endothelial markers von Willebrand factor (vWF) starting at day 14 and CD31 at day 21 (Additional file 4: Figure S2a). A significant up-regulation of vascular endothelial growth factor receptor 2 (VEGFR2) was also observed at day 28 of the differentiation (Additional file 4: Figure S2a). Unlike in undifferentiated DPPSC, at day 28, we were able to detect endothelial markers CD31, vWF and vascular endothelial cadherin (VE-cadherin) by immunofluorescence analyses in a subset of cells (Fig. 2a-c, j). The percentage of vWF^+^ VE-cadherin^+^ cells and CD31^+^ VE-cadherin^+^ was 13.7% ± 4.6% and 12.2% ± 3.3%, respectively (Fig. 2j). Finally, as endothelial differentiation progressed, cells had a tendency to form more tubular-like structures on Matrigel™ (Additional file 4: Figure S2b-d), and after 4 weeks the extent of tube formation was comparable to that of human umbilical vein endothelial cells (HUVECs; Additional file 4: Figure S2d-f).

DPPSC subjected to SMC differentiation conditions became bigger and flatter at day 4. At day 10 of differentiation, immunofluorescence analyses for α-smooth muscle actin (αSMA) and calponin revealed that differentiated DPPSC populations contained a high number of SMCs, in contrast to undifferentiated cells (Fig. 2d-f, k and Additional file 5: Figure S3a-d). We also observed that passaging or transduction with a tGFP-encoding reporter virus did not significantly affect the SMC differentiation potential of DPPSC (Fig. 2 k and Additional file 5: Figure S3d).

DPPSC cultured alone for 7 days in myogenic differentiation medium showed rare multinucleated cells positive for myosin heavy chain (MyHC; Additional file 5: Figure S3e, f). DPPSC co-cultured with C2C12 cells (a mouse myoblast cell line) revealed better quality of myotubes compared to control C2C12 cells at day 3 and 4 of myogenic induction (Additional file 5: Figure S3g-j). At day 5 of differentiation, immunofluorescence analyses showed MyHC^+^ multinucleated myotubes containing human nuclei, unlike in undifferentiated DPPSC (Fig. 2g, i and Additional file 5: Figure S3k-m). tGFP^+^ myotubes were also observed in co-cultures using tGFP^+^ DPPSC (Fig. 2h), confirming that DPPSC can fuse into myotubes. DPPSC from different donors and passages showed comparable results (Fig. 2g and Additional file 5: Figure S3k-m). Fusion index (Fig. 2l) and the number of MyHC^+^ myotubes (Fig. 2m) of the co-cultures were significantly higher compared to control C2C12 cells.

### The healing potential of DPPSC in skin wounds

**Fig. 3:**
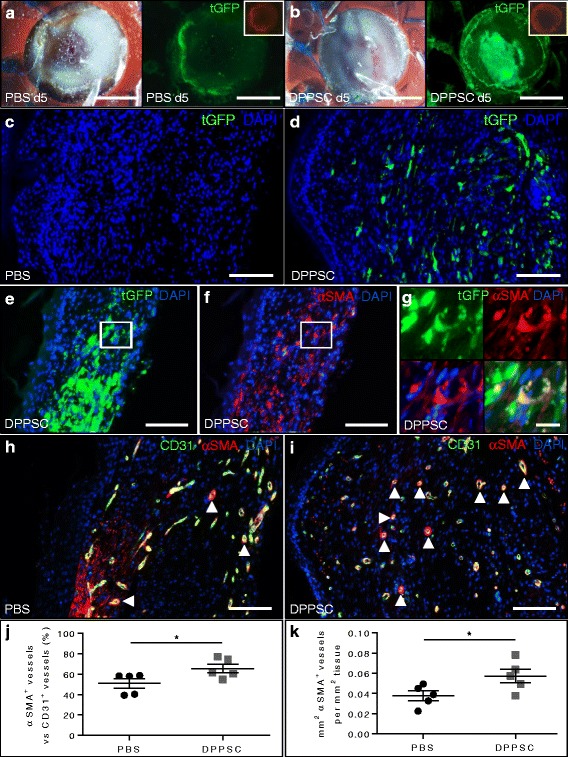
DPPSC engraftment, differentiation and revascularisation in skin wounds. **a**, **b** Bright field images and corresponding fluorescence images showing tGFP expression (*green*) in wounds (*with silicone rings*) at day 5 in mice treated with tGFP^+^ DPPSC (**b**) but not with PBS (**a**). Unspecific autofluorescence can be observed in the TRITC channel (*upper insets*). Scale bars: 5 mm. **c**, **d** Immunofluorescence analysis for tGFP (*green*) in paraffin embedded sections of wounds treated with PBS (**c**) or DPPSC (**d**) at day 11. Scale bars: 100 μm. **e**-**g**) tGFP (*green*; **e**, **g**) and αSMA (*red*; **f**, **g**) double staining on wound cross-sections reveal double-positive cells (*yellow*; **g**) representing DPPSC integrated in a vessel-like structure. Scale bars: 100 μm (**e**, **f**), 20 μm (**g**). **h**, **i** CD31 (*green*) and αSMA (*red*) immunofluorescence analysis in PBS (**h**) or DPPSC-treated (**i**) wounds, showing the presence of CD31^+^ vessels with αSMA coverage (*arrowheads*). Scale bars: 100 μm. For **c**-**i**, nuclei are counterstained with DAPI (*blue*). **j** Quantification of αSMA-coated vessels in PBS and DPPSC-treated wounds, showing higher percentage of coverage in DPPSC-treated wounds. *p < 0.05, *n* = 5 mice/group. **k** Quantification of the area of αSMA-coated vessels in PBS and DPPSC-treated wounds, showing a larger area of coverage in DPPSC-treated wounds. *p < 0.05, *n* = 5 mice/group. For **j**, **k**, two-tailed Student’s *t* test was used and results are displayed as mean ± s.e.m

**Fig. 4:**
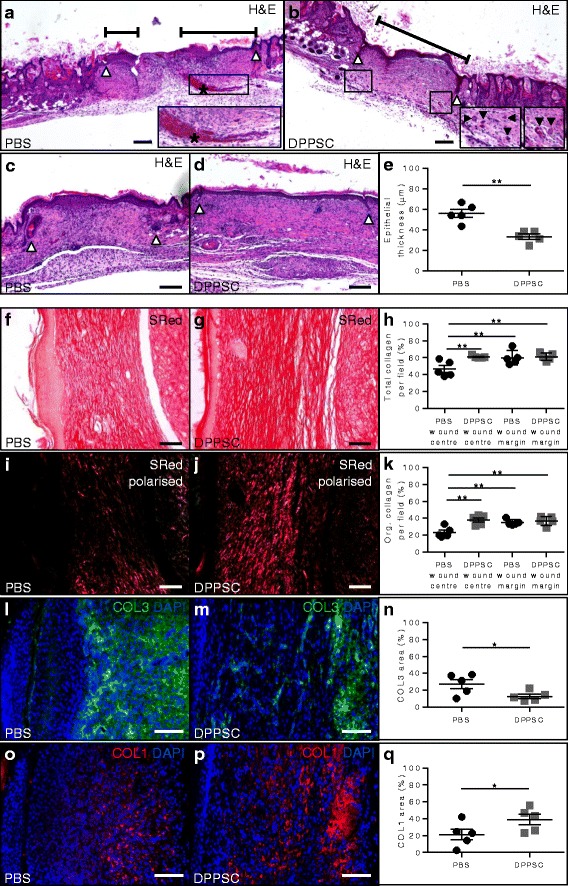
Effect of DPPSC on epidermal and dermal healing at day 11. **a**, **b** Haematoxylin and eosin staining showing the increased epithelial coverage in DPPSC-treated wounds (**b**) compared to PBS-treated ones (**a**). Wound boundaries and the length covered by the epidermis are indicated by *white arrowheads* and *black lines*, respectively. Presence of red blood cell (RBC) leakage (indicated by *asterisk* in **a**, *enlarged in inset*) can be observed in the PBS-treated condition, while RBCs are contained in blood vessels in DPPSC-treated wounds (**b**, *insets*). Scale bars: 200 μm. **c**, **d** Haematoxylin and eosin staining showing the increased epithelial thickness in the wound area (wound boundaries are indicated by *white arrowheads*) in PBS-treated mice (**c**) compared to DPPSC-treated mice (**d**). Scale bars: 200 μm. **e** Quantification of the epithelial thickness in μm. ** *p* < 0.01, *n* = 5 mice/group. **f**, **g** Sirius *Red* staining visualised by bright field microscopy of the central part of wounds treated with PBS (**f**) or DPPSC (**g**) for the analysis of total collagen in the wound matrix. Scale bars: 100 μm. **h** Quantification of the total collagen present in the wound centre and margins of mice treated with PBS or DPPSC. **p < 0.01, *n* = 5 mice/group. **i**, **j** Sirius Red staining of the same sections of wounds treated with PBS (**i**) or DPPSC (**j**) as in **f**, **g** visualised by polarised light microscopy for the analysis of organised (red birefringent) collagen in the wound matrix. Scale bars: 100 μm. **k** Quantification of the organised collagen present in the wound centre and margins of mice treated with PBS or DPPSC. **p < 0.01, *n* = 5 mice/group. **l**, **m** Immunofluorescence analysis for collagen type III (COL3; in *green*) in wounds treated with PBS (**l**) or DPPSC (**m**). **n** Quantification of the percentage of wound area positive for COL3 I in the wound centre of mice treated with PBS or DPPSC. *p < 0.05, *n* = 5 mice/group. **o**, **p** Immunofluorescence analysis for collagen type I (COL1; in *red*) in wounds treated with PBS (**o**) or DPPSC (**p**). **q** Quantification of the percentage of wound area positive for COL1 in the wound centre of mice treated with PBS or DPPSC. *p < 0.05, *n* = 5 mice/group. For **l**, **m**, **o**, **p**, nuclei are counterstained with DAPI (*blue*); scale bars: 100 μm. Two-tailed Student’s *t* test was used for **e**, **n**, **q**; one-way ANOVA test was used for **h**, **k**; results are displayed as mean ± s.e.m

Full-thickness wounds were made on the back of nude *Foxn1* mice, splinted with silicone rings, treated with PBS or tGFP^+^ DPPSC (*n* = 5 per group) and covered with a bandage to prevent the wounds from drying out. The homogeneous spreading of the cells across the wound bed was confirmed at day 5 using live fluorescence microscopy (Fig. 3a, b). Digital pictures of the wounds revealed no differences in wound contraction between treatment regimens (data not shown). At day 11 after wounding, mice were sacrificed and persistent tGFP^+^ DPPSC were detected on wound cross-sections (Fig. 3c, d). Immunofluorescence analyses for tGFP and αSMA (Fig. 3e-g) revealed that DPPSC were distributed across the regenerating area and that some DPPSC were integrated in the αSMA layer of red blood cell (RBC)-filled blood vessels, consistent with their robust in vitro SMC differentiation potential. Subsequently, immunofluorescence analysis for CD31 and αSMA (Fig. 3 h, i) revealed that, while no significant differences were found between the two groups regarding number and area of CD31^+^ vessels (Table 1), the area and percentage of αSMA-coated blood vessels were statistically significantly higher in DPPSC-treated compared to PBS-treated wounds (Fig. 3j, k and Table 1). In addition, in agreement with this improved SMC-coverage of the newly formed vessels, we observed that DPPSC-treated wounds presented less RBC leakage compared to controls (Fig. 4a, b and Additional file 6: Figure S4a-d). Laminin immunofluorescence staining for the analysis of the basement membrane of the endothelium was comparable in PBS-treated and DPPSC-treated wounds (Table 1 and Additional file S6: Figure S4e-f).

**Table 1:** Effects of DPPSC treatment in the wound healing assay

Wound revascularisation, epidermal and dermal healing at day 11			PBS	DPPSC
Revascularisation	Number of CD31^+^ vessels/mm^2^ tissue		461 ± 49	409 ± 42
	mm^2^ CD31^+^ vessels/mm^2^ tissue		0.07 ± 0.01	0.08 ± 0.02
	αSMA-coated vessels vs CD31^+^ vessels (%)		51 ± 5	66 ± 4*
	mm^2^ αSMA-coated vessels/mm^2^ tissue		0.04 ± 0.01	0.06 ± 0.01*
	RBC leakage		High	Low
	Laminin (%)		4.1 ± 1.0	4.8 ± 1.0
Epidermal healing	Mice with complete epithelial coverage (%)		40	100
	Epithelial coverage (%)		82 ± 10	100 ± 0
	Epithelial thickness (μm)		56 ± 4	33 ± 3**
Dermal healing	Total collagen (%)	wound centre	47 ± 4	61 ± 1**
		wound margin	60 ± 4	61 ± 2
	Organised collagen (%)	wound centre	23 ± 3	38 ± 3**
		wound margin	35 ± 1	37 ± 2
	Collagen type III (%)	wound centre	27 ± 5	12 ± 3*
	Collagen type I (%)	wound centre	20 ± 5	39 ± 6*
	tGFP^+^ αSMA^+^ cells not associated to vessels (%)		0 ± 0	1.3 ± 0.7

*DPPSC* dental pulp pluripotent-like stem cells, αSMA α-smooth muscle actin, *RBC* red blood cell, *tGFP* turbo green fluorescent protein

*p < 0.05, **p < 0.01 vs PBS treatment, *n* = 5 animals/group. Results are displayed as mean ± s.e.m.

Next, we evaluated the benefit of DPPSC transplantation on epidermal healing by analysing re-epithelialisation. While only 40% of the PBS-treated mice showed complete epithelial coverage of the wound, all DPPSC-treated mice had complete re-epithelialisation (Table 1). In those PBS-treated mice that were incompletely re-epithelialised, the average coverage was 70% ± 10% (Fig. 4a, b). In addition, the thickness of the epithelium in DPPSC-treated wounds compared to that of PBS-treated wounds was significantly lower, resembling that of normal skin outside the wound (Fig. 4c-e and Table 1). To evaluate dermal healing we analysed the deposition and organisation of collagen in the wound borders and centre. The collagen content was statistically significantly higher in the centre of the DPPSC-treated wounds compared to the PBS-treated wounds as indicated by Sirius Red staining under bright light (Fig. 4f-h and Table 1). The wound margins adjacent to the normal skin showed no significant difference between the two groups (Fig. 4 h and Table 1). Furthermore, the amount of organised (red birefringent) collagen, revealed with polarised light microscopy on the same cross-sections, was also significantly higher in the centre of the DPPSC-treated compared to the PBS-treated wounds (Fig. 4i-k and Table 1) and no statistically significant differences were observed in the wound margins (Fig. 4k and Table 1). Interestingly, unlike in PBS-treated animals, the values of total and organised collagen in the centre of the DPPSC-treated wounds were similar to the ones in the wound margins and normal skin tissue (Fig. 4h, k). These differences in collagen organisation in the centre of the wounds were confirmed by immunofluorescence analyses for collagen type III (COL3), representing firstly formed more disorganised fibres, and collagen type I (COL1), representing later formed more organised fibres (Fig. 4l-q and Table 1). Finally, DPPSC-derived myofibroblasts (αSMA^+^tGFP^+^ cells not associated to vessel structures) were barely detected in the wound area (Table 1 and Additional file 6: Figure S4g, h).

### The healing potential of DPPSC in dystrophic muscles

**Fig. 5:**
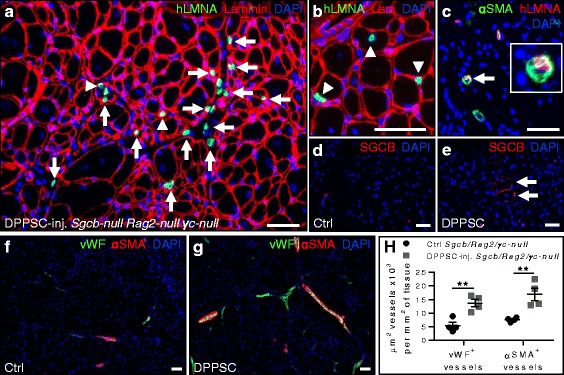
DPPSC engraftment, differentiation and revascularisation in *Sgcb-null Rag2-null γc-null* dystrophic mice at day 20. **a**, **b** Immunofluorescence analysis for hLMNA (*green)* and laminin (*red*), showing DPPSC engraftment in *Sgcb-null Rag2-null γc-null* mice. *Arrows* indicate DPPSC in the interstitial space, while *arrowheads* indicate integration in the fibres. **c** Immunofluorescence analysis for hLMNA (*red*) and αSMA (*green*) in DPPSC-injected *Sgcb-null Rag2-null γc-null* muscles; a higher magnification of a human cell integrated in the SMC layer of a blood vessel is shown in the inset. **d**, **e** Immunofluorescence analysis for beta-sarcoglycan (SGCB; in *red*) in control (**d**) and DPPSC-injected (**e**) *Sgcb-null Rag2-null γc-null* muscles. **f**, **g** vWF (*green*) and αSMA (*red*) immunofluorescence analysis in control (**f**) or DPPSC-injected (**g**) *Sgcb-null Rag2-null γc-null* muscles, showing the presence of vWF^+^ vessels with αSMA coverage. For **a**-**g**, nuclei are counterstained with DAPI (*blue*); scale bars: 50 μm. **h** Quantitative analysis of the area of vWF^+^ or αSMA^+^ vessels per mm^2^ of tissue in *Sgcb-null Rag2-null γc-null* muscles. **p < 0.01, *n* = 4 mice/group. Two-tailed Student’s *t* test was used and results are displayed as mean ± s.e.m

DPPSC were injected in the tibialis anterior of *Sgcb-null Rag2-null γc-null* and *Scid/mdx* mice. In *Sgcb-null Rag2-null γc-null* mice, DPPSC engraftment in muscle tissue 20 days after the injection was confirmed by immunofluorescence analysis for human lamin A/C (hLMNA) and laminin (Fig. 5a). The majority of DPPSC were detected in the interstitial space among fibres, although human nuclei were occasionally present in regenerating fibres (Fig. 5a, b). In addition, DPPSC were integrated in SMC-coated blood vessels and were expressing αSMA (Fig. 5c). SGCB^+^ fibres were detected in DPPSC-injected muscles in *Sgcb-null Rag2-null γc-null* mice, while no SGCB^+^ fibres were detected in control muscles (Fig. 5d, e). Quantification of the area of vessels positive for vWF and αSMA revealed that DPPSC-injected muscles had a higher area of vWF^+^ or αSMA^+^ vessels compared to control muscles in *Sgcb-null Rag2-null γc-null* mice (Fig. 5f-h).

In *Scid/mdx*, DPPSC engraftment was also confirmed (Additional file 7: Figure S5a) and human nuclei were mainly localised in the interstitial space and occasionally in regenerating fibres (Additional file 7: Figure S5a-c). Moreover, vWF^+^ DPPSC were detected in the EC layer and αSMA^+^ DPPSC in the SMC layer of blood vessels (Additional file 7: Figure S7d, e). In DPPSC-injected muscles*,* more dystrophin^+^ fibres were observed compared to control muscles (Additional file 7: Figure S5f, g). We could also detect, in serial sections, dystrophin expression in the same zones where hLMNA^+^ DPPSC were present (Additional file 7: Figure S5h, i), suggesting their direct myogenic contribution. The area of vWF^+^ or αSMA^+^ vessels in DPPSC-injected muscles in *Scid/mdx* was also higher compared to control muscles (Additional file 7: Figure S5j-l).

**Fig. 6:**
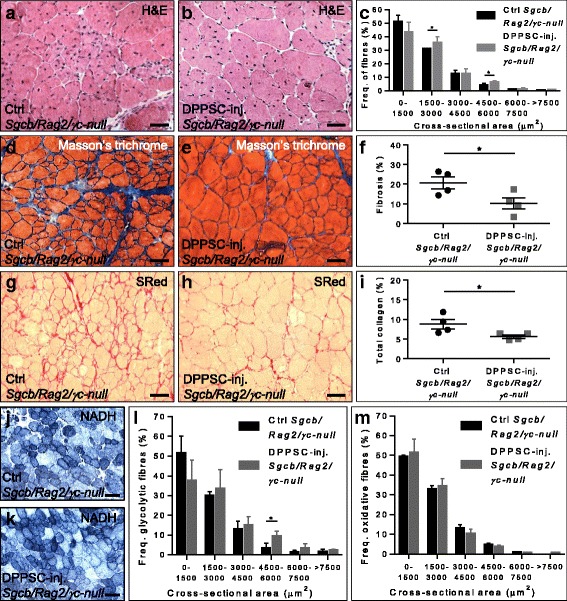
Histological, morphometric and fibre type analyses after DPPSC injection in dystrophic *Sgcb-null Rag2-null γc-null* mice. **a**, **b** Haematoxylin and eosin staining in control (**a**) or DPPSC-injected (**b**) *Sgcb-null Rag2-null γc-null* muscles. Scale bars: 100 μm. **c** Quantitative frequency distribution analysis of the cross-sectional area of the fibres in *Sgcb-null Rag2-null γc-null* muscles. *p < 0.05, *n* = 3 for each group. **d**, **e** Masson’s trichrome staining in control (**d**) or DPPSC-injected (**e**) *Sgcb-null Rag2-null γc-null* muscles revealing areas of fibrosis (*blue*). Scale bars: 100 μm. **f** Quantitative analysis of the percentage of fibrosis per field in *Sgcb-null Rag2-null γc-null* muscles. *p < 0.05, *n* = 4 for each group. **g**, **h** Sirius Red staining visualised by bright field microscopy in control (**g**) or DPPSC-injected (**h**) *Sgcb-null Rag2-null γc-null* muscles for the analysis of total collagen. Scale bars: 100 μm. **i** Quantification of the total collagen present in *Sgcb-null Rag2-null γc-null* muscles. *p < 0.05, *n* = 4 for each group. **j**, **k** NADH staining of control (**j**) and DPPSC-injected (**k**) *Sgcb-null Rag2-null γc-null* muscles, showing oxidative fibres in *blue* and glycolytic fibres in *white*. Scale bars: 100 μm. **l**, **m** Quantitative frequency distribution analysis of the cross-sectional area of type II fast-glycolytic (**l**) or type I slow-oxidative (**m**) fibres in the NADH staining in *Sgcb-null Rag2-null γc-null* muscles injected with DPPSC compared to control muscles. *p < 0.05, *n* = 3 for each group. For **c**, **f**, **i**, **l**, **m**, two-tailed Student’s *t* test was used and results are displayed as mean ± s.e.m

Morphometric analysis of muscle tissue revealed that DPPSC-injected muscles featured a higher frequency of fibres with larger cross-sectional area when compared to control muscles in both mouse models (Fig. 6a-c and Additional file 8: Figure S6a-c). Moreover, Masson’s trichrome staining showed a significant reduction in fibrosis in DPPSC-injected dystrophic muscles compared to controls (Fig. 6d-f and Additional file 8: Figure S6d-f). This was confirmed by Sirius Red staining (Fig. 6 g-i and Additional file 8: Figure S6g-i), that revealed significantly lower collagen content in DPPSC-injected muscles compared to controls.

Interestingly, NADH staining showed that in *Sgcb-null Rag2-null γc-null* muscles larger type II fast-glycolytic fibres were observed in DPPSC-injected muscles compared to control muscles (Fig. 6j-l). Type I slow-oxidative fibres were comparable in *Sgcb-null Rag2-null γc-null* muscles injected with DPPSC and in control muscles (Fig. 6j, k, m). In *Scid/mdx* mice, however, DPPSC-injected muscles featured larger type I and type II fibres compared to control muscles (Additional file 8: Figure S6j-m).

**Fig. 7:**
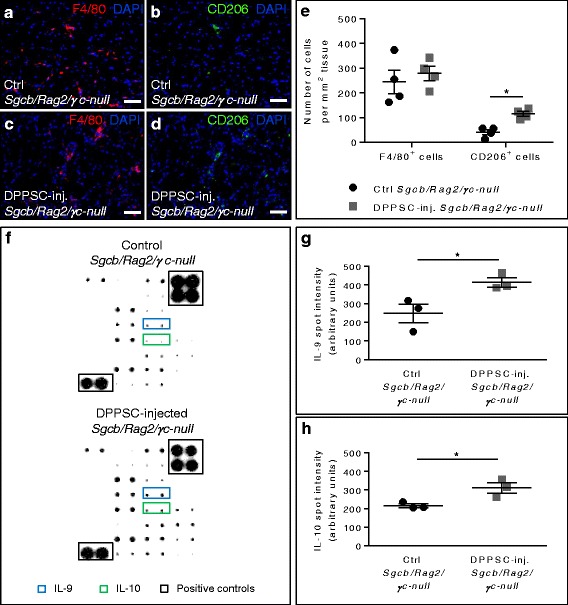
Macrophage and cytokine analyses after DPPSC injection in dystrophic *Sgcb-null Rag2-null γc-null* mice. **a**-**d** Immunofluorescence analysis for macrophage-specific F4/80 (*red*; **a**, **c**) and proangiogenic M2 macrophage-specific CD206 (*green*; **b**, **d**) in control (**a**, **b**) or DPPSC-injected (**c**, **d**) *Sgcb-null Rag2-null γc-null* muscles. Scale bars: 50 μm. **e** Quantitative analysis of the number of cells expressing F4/80 and CD206 macrophage markers per mm^2^ of tissue in *Sgcb-null Rag2-null γc-null* muscles. *p < 0.05, *n* = 4 for each group. **f** Representative images of cytokine antibody arrays showing the increase in spot intensity in IL-9 and IL-10 in DPPSC-injected (*lower panel*) compared to control (*upper panel*) *Sgcb-null Rag2-null γc-null* muscles. **g**, **h** Quantitative analysis of the increase in spot intensity (arbitrary units) in IL-9 (**g**) and IL-10 (**h**) in *Sgcb-null Rag2-null γc-null* muscles. *p < 0.05, *n* = 3 for each group. For **e**, **g**, **h**, two-tailed Student’s *t* test was used and results are displayed as mean ± s.e.m

In order to analyse the effect of DPPSC on macrophage polarisation, we performed immunofluorescence analyses for the general and proangiogenic M2 macrophage markers F4/80 and CD206, respectively. In both mouse models DPPSC treatment did not alter the total amount of F4/80^+^ macrophages (Fig. 7a, c, e and Additional file 9: Figure S7 a, c, e). However, the amount of CD206^+^ cells was significantly higher in DPPSC-injected compared to control muscles in both mouse models (Fig. 7b, d, e and Additional file 9: Figure S7b, d, e). To verify whether the change of macrophage-specific markers was associated with cytokine profile modifications, we analysed the presence of cytokines in DPPSC-injected and control muscles. Our data showed that several factors involved in M2 transition, including interleukin (IL)-4, IL-5, IL-6 and GCSF were not influenced by DPPSC treatment. However, the increased amount of IL-9 and IL-10 in DPPSC-injected muscles compared to control muscles (Fig. 7f-h and Additional file 9: Figure S7f) suggested that these cytokines could be implicated in M2 transition.

## Discussion

In this study, we isolated several DPPSC populations as previously reported [1, 2, 31]. DPPSC maintained their typical morphology for 15 passages, i.e.*,* small cells with large nuclei relative to the volume of the cytoplasm, a characteristic shared with other hPSCs [32]. In addition, DPPSC expressed pluripotency markers, including OCT4. The detection of OCT4 expression by PCR has been controversial, due to the presence of transcriptional variants of the *OCT4* gene and pseudogenes that encode proteins that do not participate in pluripotency maintenance [33, 34]. Therefore, for our analyses, we used the primers from Xu et al. [33] that specifically amplify the mRNA variant 1 or *OCT4A*, which synthesises the key transcription factor for maintaining self-renewal and pluripotency. The presence of NANOG and SOX2 proteins was also confirmed by immunofluorescence analysis. DPPSC were also characterised by the presence of ALP activity, which is known to be present in adult and pluripotent stem cells [35, 36]. A main concern for potential clinical application based on stem cell therapy is the genetic stability [37, 38]. In this study, by using the sCGH technique [30], we confirmed that DPPSC from ten different donors showed no chromosomal abnormalities for at least 15 passages.

Functional blood vessels consist of mainly two distinct cell types, i.e.*,* ECs (lining the inside) and vascular SMCs (surrounding the ECs) [3]. We provided evidence that DPPSC differentiate into functional ECs expressing typical endothelial markers (VEGFR2, CD31, VE-cadherin and vWF) at mRNA and protein levels. The addition of BMP4, an inducer of mesoderm, in the first days of the differentiation [39, 40] could be further explored to optimise the endothelial potential of DPPSC. The percentages of differentiated cells that expressed typical SMC markers were higher as compared to EC differentiation. In addition, undifferentiated DPPSC maintained the SMC differentiation capacity up to passage 10. During embryonic development, ectomesenchymal cells in the cranial neural folds have the competence to form SMCs [41, 42]. Thus, the capacity of DPPSC to undergo SMC differentiation could be related to their neuroectodermal differentiation potential and is extremely important for in vivo applications where new functional blood vessels are needed.

To analyse the revascularisation potential of DPPSC in vivo*,* we first used a full thickness wound healing assay described by Hendrickx et al*.* [18] and in addition studied the effect on wound closure and (epi)dermal healing after 11 days. DPPSC were shown for the first time to differentiate into perivascular SMCs, demonstrating a direct contribution to vascular SMC coating of blood vessels. In addition, a significant increase in the percentage and area of mature αSMA^+^-coated vessels was found in the healing wound bed of DPPSC-treated mice compared to controls. This could be likely due to a paracrine effect of DPPSC, able to secrete growth factors such as ANG, VEGF, PDGF-BB, HGF, PlGF and osteopontin (OPN). Indeed, PDGF-BB, PlGF and OPN are known to attract SMCs and stabilise vessels [43–46]. In agreement with a more stable wound vasculature, granulation tissue in DPPSC-treated wounds featured less RBC leakage. Other studies reported that intramyocardial injection of human dental pulp stem cells (DPSCs) in a rat model of myocardial infarction, resulted in a better neovascularisation in cell-treated animals compared to controls and an improvement in cardiac function [47]. Along with an increased maturation of the wound vasculature, we here demonstrated that all DPPSC-treated wounds showed complete epithelial coverage in contrast to only 40% of PBS-treated wounds and that the average epithelial thickness was significantly lower in DPPSC-treated wounds, suggesting that both the epithelial coverage and the subsequent involution of the hyperplastic neoepidermis were accelerated in DPPSC-treated mice. Our novel data regarding DPPSC secretion support an indirect mechanism for the accelerated epithelial coverage mediated by the angiogenic factors VEGF, ANG and PDGF-BB that improved oxygen delivery to migrating keratinocytes. In addition, DPPSC secreted a huge amount of HGF and TIMP-2 (and a tiny amount of IL-6), which have been shown to promote keratinocyte migration [48–50]. In further support of a more mature and advanced status of the wound healing process in DPPSC-treated mice, dermal wound collagen deposition was significantly higher in the centre of DPPSC-treated wounds, better resembling the characteristics of normal skin. It is likely that the growth factors secreted by DPPSC could be responsible at least in part for the beneficial effect of DPPSC treatment in the collagen deposition. Indeed, DPPSC production of TGF-β could promote fibroblast proliferation and collagen synthesis [17].

DPPSC injected in dystrophic muscles differentiated into both ECs and SMCs. Indeed, DPPSC were detected in the inner and outer part of vessels in dystrophic *Scid/mdx* mice. In dystrophic *Sgcb-null Rag2-null γc-null* mice, cells were often detected in the outer part of vessels, also suggesting a significant involvement in revascularisation. In a recent study [28], it was observed that human DPSCs, even though they were pre-differentiated towards the skeletal muscle lineage, were localised in the endothelium of newly generated vessels after their transplantation in *Scid/mdx* mice. We quantified the area of vessels present in the muscles and found that DPPSC-injected muscles had a significantly higher area of vWF^+^ and of αSMA^+^ vessels compared to control muscles, further supporting the revascularisation potential of DPPSC. Intriguingly, while DPPSC increased the SMC coverage rate but not the total number of blood vessels in healing wounds, they increased the total number of vessels in both dystrophic models. We speculate this differential revascularisation response may be in part related to a different time range of both healing processes, the duration being twice as long in the dystrophic models. The longer duration of the latter would be permissive for the longer term required for DPPSC to differentiate in vitro into mature ECs as compared to SMCs. In addition, the fact that the efficiency of DPPSC in vitro EC differentiation was not as high as SMC differentiation could also partially explain their differential revascularisation response.

In this study, we also provided evidence that DPPSC from different donors are capable of fusing with mouse C2C12 cells after 5 days of myogenic induction, thereby generating hybrid myotubes. The capacity to fuse was maintained for at least ten passages. We also observed that the presence of DPPSC enhanced the differentiation potential of C2C12 cells, increasing the fusion index and the number of MyHC^+^ myotubes. Consistent with our results, a recent study using DPSCs demonstrated that DPSCs are able to fuse with C2C12 cells forming hybrid myotubes, although myogenic induction was prolonged to 14 days [28]. Similarly to DPSCs, DPPSC contributed to myogenic regeneration when transplanted in *Scid/mdx* mice. Moreover, the results presented here demonstrated for the first time that DPPSC can also contribute to regeneration of muscle fibres in vivo when injected intramuscularly in *Sgcb-null* dystrophic mice. This is of clinical relevance since in contrast to *mdx* mice, *Sgcb-null* mice do not feature revertant fibres and thus exhibit severe muscular dystrophy, better replicating the human disease [23, 25, 51]. We also detected the presence of beta-sarcoglycan^+^ fibres in DPPSC-injected *Sgcb-null Rag2-null γc-null* mice. In addition, dystrophic muscles from both murine models injected with DPPSC featured a higher frequency of fibres with larger cross-sectional area and reduced fibrosis and collagen content, expanding the previous report in *Scid/mdx* mice using DPSCs [28] also for the limb-girdle muscular dystrophy mouse model *Sgcb-null Rag2-null γc-null*. Moreover, DPPSC-injected dystrophic muscles showed a higher frequency of type II fast-glycolytic fibres with higher cross-sectional area compared to controls in both dystrophic mouse models. This is particularly relevant since it has been proven that fast glycolytic muscle fibres are preferentially affected in DMD [52] and that at least in *mdx* mice they are highly susceptible to degeneration-regeneration cycles [53, 54]. Therefore, we hypothesise that DPPSC play a role in reducing the degeneration of glycolytic fibres in these mice. Mechanistic insights to explain these beneficial effects are still unknown and further experiments are needed to address this issue. Nevertheless, we hypothesise that the detected growth factors secreted by DPPSC could play a synergistic role in the positive impact of DPPSC on dystrophic muscle. For instance, HGF is considered the only growth factor able to activate satellite cells, the muscle-resident myogenic stem cells [55]. In addition, DPPSC injection in dystrophic skeletal muscles also resulted in an increase of CD206^+^ cells compared to control muscles, while the total number of F4/80^+^ cells was maintained. F4/80 is a well-known macrophage-specific marker [56] and CD206 (mannose receptor) has been used to distinguish M2 macrophages with proangiogenic [57] and anti-inflammatory properties [58] from pro-inflammatory M1 macrophages. Thus, the higher presence of CD206^+^ cells in DPPSC-injected muscles further explained the beneficial effect of DPPSC treatment on blood vessel growth and prevention of muscle degeneration. Unfortunately, xenograft models imply the use of immunodeficient animals that are a clear limitation to investigate cellular and molecular mechanisms connected to the immune system and likely in part responsible for the beneficial effect of DPPSC. In previous studies, other adult stem cells have shown the capacity to activate M2 macrophages [59]. Similarly to those studies, it is likely that in DPPSC-treated muscles M2 macrophages were engaged through paracrine signalling. Interestingly, DPPSC-injected muscles presented significantly higher levels of IL-9 and IL-10 compared to controls. Indeed, several reports have shown that IL-10 is primarily involved in the M2 transition and promotes muscle regeneration after acute and chronic injury [60, 61]. However, in our experiments, IL-13, also implicated in M2 transition, was only slightly affected. Finally, IL-9 still remains an understudied cytokine [62]. Nevertheless, it is known to be mainly produced in response to the M2-related cytokines IL-4 and TGF-β [63] and to promote progenitor cell proliferation and reduce apoptosis [64], thus probably contributing to a better regeneration in DPPSC-injected muscles.

## Conclusions

Taken together, our results showed that DPPSC positively impact on dystrophic skeletal muscles and wound healing assays. Although our findings are not conclusive to determine the modus operandi of transplanted DPPSC, it is clear that they act mainly through their revascularisation potential, paracrine signalling effects and limited smooth and skeletal muscle direct contribution. Further studies are necessary to test whether these actions could functionally improve muscle histopathologies.

## Additional files

Additional file 1: Table S1. List of primers used for cDNA amplification in qRT-PCR analyses. (DOCX 11 kb)

Additional file 2: Table S2. List of antibodies used for protein detection in immunofluorescence analyses. (DOCX 16 kb)

Additional file 3: Figure S1. DPPSC characterisation. **a**-**d** Phase contrast images of DPPSC morphology in the primary culture (**a**), passage (P)5 (**b**), P10 (**c**) and P15 (**d**). Scale bars: 200 μm. **e** Population-doubling time in hours of DPPSC for 15 passages. *n* = 13 different donors, results are displayed as mean ± s.e.m.. **f** Number of divisions per passage of DPPSCs for 15 passages. *n* = 13 different donors, results are displayed as mean ± s.e.m. **g** Example of a short-Comparative Genomic Hybridisation in DPPSC at P15 showing no chromosomal abnormalities. The DNA control used for the hybridisation was XXY, therefore the observed loss of chromosome Y indicates these cells are from a female donor. **h** Relative mRNA fold change expression of the pluripotency markers *OCT4A* and *NANOG* at P5, P10 and P15 compared to P5 in DPPSC from eight different donors. ***p < 0.001, *n* = 8 different donors, two-way ANOVA was used and results are displayed as mean ± s.e.m. **i**, **j** Immunofluorescence analyses for the pluripotency markers NANOG (*red*; **i**) and SOX2 (*red*; **j**) in undifferentiated DPPSC at P10. Nuclei are counterstained with DAPI (*blue*). Merge is observed in *purple*. Scale bars: 100 μm. **k** Alkaline phosphatase (ALP) staining of DPPSC at P10. Scale bar: 100 μm. (PDF 196 kb)

Additional file 4: Figure S2. In vitro endothelial differentiation of DPPSC. **a** qRT-PCR of the endothelial markers *VEGFR2*, *CD31* and *vWF* at different differentiation time points. HUVECs were used as controls and *GAPDH* as housekeeping gene. *p < 0.05, **p < 0.01, ***p < 0.001, ^#^ *p* < 0.05 (*vWF* gene expression d0 vs d14; d0 vs d28), &*p* < 0.05 (*CD31* gene expression d7 vs d14), &&&*p* < 0.001 (*vWF* gene expression d7 vs d14), *n* = 3 independent experiments, one-way ANOVA was used, results are displayed as mean ± s.e.m.. **b**-**e** Functional 2D Matrigel™ assay at 24 hours showing tube-like structures formed by DPPSC at day 14 (**b**), 21 (**c**) and 28 (**d**) of endothelial differentiation and by HUVECs (**e**). Scale bars: 500 μm. **f** Quantitative analysis of the tubular-like structures formed in the Matrigel™ assay by HUVECs and differentiated DPPSC at different time points. Data obtained by differentiated DPPSC at day 28 are statistically significant compared to those obtained in the other time points. **p < 0.01, *n* = 3 independent experiments, one-way ANOVA was used, results are displayed as mean ± s.e.m. (PDF 113 kb)

Additional file 5: Figure S3. In vitro smooth muscle and myogenic differentiation of DPPSC. **a**-**c** Immunofluorescence analysis for the smooth muscle markers αSMA (*red*) and calponin (*green*) in DPPSC from a different donor than Fig. 2d, e cultured in differentiation medium for 10 days at P5 (**a**) and P10 (**b**). tGFP^+^ DPPSC from the same donor at P10 were also analysed (**c**). Double-positive cells are shown in *orange*. Nuclei are counterstained with DAPI (*blue*). Scale bars: 100 μm. **d** Quantitative analysis of the percentage of cells expressing both smooth muscle markers (*orange*), calponin alone (*green*), αSMA alone (*red*) or none of these markers (*blue*), showing no statistically significant difference. *n* = 3 independent experiments, one-way ANOVA was used, results are displayed as mean ± s.e.m.. **e** Immunofluorescence of DPPSC differentiated for 7 days to skeletal muscle. MyHC is shown in *red* and hLMNA in *green. Yellow* lines show the presence of myotubes with more than one nucleus inside. Nuclei are counterstained with DAPI (blue). **f** Immunofluorescence of the mouse myoblast cell line C2C12 differentiated for 7 days to skeletal muscle. MyHC is shown in *red*. An example of a myotube is also indicated with yellow lines. Nuclei are counterstained with DAPI (blue). **g**-**j** Co-cultures of DPPSC-C2C12 (**g**, **i**) or C2C12 mono-cultures (**h**, **j**) at day 3 (**g**, **h**) or 4 (**i**, **j**) of skeletal muscle differentiation. **k** DPPSC from the same donor as Fig. 2g at P10 co-cultured with C2C12 cells for 5 days. *Arrows* indicate the presence of human nuclei (stained for hLMNA in *green*) inside the formed myotubes expressing MyHC (*red*). Nuclei are counterstained with DAPI (blue). **l**, **m** DPPSC from another donor at P5 (**l**) and P10 (**m**) co-cultured with C2C12 cells for 5 days. Arrows indicate the presence of human nuclei (stained for hLMNA in *green*) inside the formed myotubes expressing MyHC (*red*). Nuclei are counterstained with DAPI (*blue*). For **e**-**m**, scale bars: 100 μm. (PDF 344 kb)

Additional file 6: Figure S4. Effect of DPPSC on leakage and dermal healing. **a**, **b** Haematoxylin and eosin staining of paraffin sections of the wound tissue in PBS-treated wounds. Presence of red blood cell (RBC) leakage (indicated by *asterisks*) can be observed. Scale bars: 100 μm. **c**, **d** CD31 (*green*) and αSMA (*red*) immunofluorescence analysis in serial sections of **a**, **b**, showing the presence of autofluorescent RBC outside of the CD31^+^ vessels in PBS-treated wounds. RBC leakage is indicated by asterisks. **e**, **f** Laminin (*red*) immunofluorescence analysis in PBS (**e**) or DPPSC-treated (**f**) wounds for the analysis of the basement membrane of the endothelium. **g**, **h** tGFP (*green*) and αSMA (*red*) double staining on wound cross-sections of DPPSC-treated mice revealed very few double positive cells (*yellow*; **h**) not associated with vessel-like structures. For **c**-**h**, nuclei are counterstained with DAPI (*blue*); scale bars: 100 μm. (PDF 496 kb)

Additional file 7: Figure S5. DPPSC engraftment, differentiation and revascularisation in *Scid/mdx* dystrophic mice at day 20. **a**-**c** Immunofluorescence analysis for hLMNA (*green*) and laminin (*red*), showing DPPSC engraftment in *Scid/mdx* mice. *Arrows* indicate DPPSC in the interstitial space, while *arrowheads* indicate localisation in the basal lamina or integration inside the fibres. **d** Immunofluorescence analysis for hLMNA (*green*) and vWF (*red*) in DPPSC-injected *Scid/mdx*. **e** Immunofluorescence analysis for hLMNA (*red*) and αSMA (*green*) in DPPSC-injected *Scid/mdx*. **f**, **g** Immunofluorescence analysis for dystrophin (DYS; in *green*) in control (**f**) and DPPSC-injected *Scid/mdx* (**g**) muscles. **h**, **i** Immunofluorescence analyses in two serial sections for hLMNA (*green*; **h**) and dystrophin (*red*; **i**) in DPPSC-injected *Scid/mdx* mice. Bright field allows the identification of the same fibres in the two serial sections. **j**, **k** vWF (*green*) and αSMA (*red*) immunofluorescence analysis in control (**j**) or DPPSC-injected (**k**) *Scid/mdx* muscles. For **a**-**k**, nuclei are counterstained with DAPI (*blue*); scale bars: 50 μm. **l** Quantitative analysis of the area of vWF^+^ or αSMA^+^ vessels per mm^2^ of tissue in *Scid/mdx* muscles. **p < 0.01, ***p < 0.001, *n* = 9 mice/group. Two-tailed Student’s *t* test was used and results are displayed as mean ± s.e.m.. (PDF 281 kb)

Additional file 8: Figure S6. Histological, morphometric and fibre type analyses after DPPSC injection in dystrophic *Scid/mdx* mice. **a**, **b** Haematoxylin and eosin staining in control (**a**) or DPPSC-injected (**b**) *Scid/mdx* muscles. Scale bars: 100 μm. **c** Quantitative frequency distribution analysis of the cross-sectional area of the fibres in *Scid/mdx* muscles. *p < 0.05, *n* = 3 for each group. **d**, **e** Masson’s trichrome staining in control (**d**) or DPPSC-injected (**e**) *Scid/mdx* muscles revealing areas of fibrosis (*blue*). Scale bars: 100 μm. **f** Quantitative analysis of the percentage of fibrosis per field in *Scid/mdx* muscles. *p < 0.05, *n* = 9 for each group. **g**, **h** Sirius Red staining visualised by bright field microscopy in control (**g**) or DPPSC-injected (**h**) *Scid/mdx* muscles for the analysis of total collagen. Scale bars: 100 μm. **i** Quantification of the total collagen present in *Scid/mdx* muscles. ***p < 0.001, *n* = 8 for each group. **j**, **k** NADH staining of control (**j**) and DPPSC-injected (**k**) *Scid/mdx* muscles, showing oxidative fibres in *blue* and glycolytic fibres in *white*. Scale bars: 100 μm. **l**, **m** Quantitative frequency distribution analysis of the cross-sectional area of type II fast-glycolytic (**l**) or type I slow-oxidative (**m**) fibres in the NADH staining in *Scid/mdx* muscles injected with DPPSC compared to control muscles. *p < 0.05, *n* = 3 for each group. For **c**, **f**, **i**, **l**, **m**, two-tailed Student’s *t* test was used and results are displayed as mean ± s.e.m.. (PDF 310 kb)

Additional file 9: Figure S7. Macrophage and cytokine analyses after DPPSC injection in dystrophic *Scid/mdx* mice. **a**-**d** Immunofluorescence analysis of macrophage-specific F4/80 (*red*; **a**, **c**) and proangiogenic M2 macrophage-specific CD206 (*green*; **b**, **d**) in control (**a**, **b**) or DPPSC-injected (**c**, **d**) *Scid/mdx* muscles. Scale bars: 50 μm. **e** Quantitative analysis of the number of cells expressing F4/80 and CD206 macrophage markers per mm^2^ of tissue in *Scid/mdx* muscles. *p < 0.05, *n* = 9 for each group; two-tailed Student’s *t* test was used and results are displayed as mean ± s.e.m.. **f** Cytokine antibody arrays showing the apparent increment in spot intensity in IL-9, IL-10 and IL-13 in DPPSC-injected (*right panel*) compared to control (*left panel*) *Scid/mdx* muscles. (PDF 51 kb)

## Abbreviations

ALP: Alkaline phosphatase
ANG: Angiogenin
COL1: Collagen type I
COL3: Collagen type III
DMD: Duchenne muscular dystrophy
DPPSC: Dental pulp pluripotent-like stem cells
DPSC: Dental pulp stem cell
EC: Endothelial cell
EGF: Epidermal growth factor
EGM-2: Endothelial Growth Medium 2
HGF: Hepatocyte growth factor
hLMNA: Human lamin A/C
HUVEC: Human umbilical vein endothelial cell
IL: Interleukin
LGMD: Limb-girdle muscular dystrophy
LGMD2E: Limb-girdle muscular dystrophy type 2E
MCP-1: Monocyte chemoattractant protein 1
MD: Muscular dystrophy
MSC: Mesenchymal stromal cell
MyHC: Myosin heavy chain
OPN: Osteopontin
PDGF: Platelet-derived growth factor
RBC: Red blood cell
sCGH: Short-comparative genomic hybridisation
SMC: Smooth muscle cell
tGFP: Turbo green fluorescent protein
TIMP-1: Tissue inhibitor of metalloproteinases 1
TIMP-2: Tissue inhibitor of metalloproteinases 2
VE-cadherin: Vascular endothelial cadherin
VEGF: Vascular endothelial growth factor
VEGFR2: Vascular endothelial growth factor receptor 2
vWF: von Willebrand factor
αSMA: α-smooth muscle actin
