# Supplementary material for: Human dental pulp pluripotent-like stem cells promote wound healing and muscle regeneration
Source: Stem Cell Res Ther. 2017 Jul 27;8:175. doi: 10.1186/s13287-017-0621-3 (PMC5531092; doi:10.1186/s13287-017-0621-3)
Supplement: Supplementary file 1 — List of primers used for cDNA amplification in qRT-PCR analyses. (DOCX 11 kb) [file 13287_2017_621_MOESM1_ESM.docx]

**Table S1:** List of primers used for cDNA amplification in the qRT-PCR analyses.

| **Gene** | **Forward** | **Reverse** |
| --- | --- | --- |
| ***OCT4A*** | *CTTCGCAAGCCCTCATTTCACC* | *CCAGGTCCGAGGATCAACC* |
| ***NANOG*** | *AACAGGTGAAGACCTGGTTCC* | *CTGAGGCCTTCTGCGTCACA* |
| ***GAPDH*** | *CTGGTAAAGTGGATATTGTTGCCAT* | *TGGAATCATATTGGAACATGTAAACC* |
| ***VEGFR2*** | *TGG CAT CGC GAA AGT GTA TC* | *AAA GGG AGG CGA GCA TCT C* |
| ***CD31*** | *ACT GCA CAG CCT TCA ACA GA* | *TTT CTT CCA TGG GGC AAG* |
| ***vWF*** | *GTC GAG CTG CAC AGT GAC AT* | *CCA CGT AAG GAA CAG AGA CCA* |
