# Supplementary material for: Human dental pulp pluripotent-like stem cells promote wound healing and muscle regeneration
Source: Stem Cell Res Ther. 2017 Jul 27;8:175. doi: 10.1186/s13287-017-0621-3 (PMC5531092; doi:10.1186/s13287-017-0621-3)
Supplement: Supplementary file 2 — List of antibodies used for protein detection in immunofluorescence analyses. (DOCX 16 kb) [file 13287_2017_621_MOESM2_ESM.docx]

**Table S2:** List of antibodies used for protein detection in immunofluorescence analyses.

| **Antibody against** | **Company** | **Cat. Number** | **Dilution** |
| --- | --- | --- | --- |
| **NANOG** | Abcam | ab80892 | 1:100 |
| **SOX2** | Santa Cruz Biotechnology | sc-17320 | 1:50 |
| **VE-CADHERIN** | Santa Cruz Biotechnology | sc-9989 | 1:50 |
| **CD31** | Millipore  BD Biosciences | 04-1074  BD557355 | 1:100  1:500 |
| **vWF** | Abcam | ab6994 | 1:400 |
| **CALPONIN** | Abcam | ab46794 | 1:200 |
| **αSMA** | Sigma | C6198  A2547 | 1:200  1:400 |
| **hLMNA** | Epitomics  Novocastra | 2966-1  NCL-LAM-A/C | 1:600  1:100 |
| **MyHC** | Hybridoma Bank | - | 1:5 |
| **tGFP** | Evrogen | AB513 | 1:500 |
| **Laminin** | Sigma | L9393 | 1:300 |
| **COL 1** | Abcam | ab34710 | 1:100 |
| **COL 3** | Abcam | ab7778 | 1:400 |
| **SGCB** | Novocastra | NCL-b-SARC | 1:75 |
| **DYS** | Novocastra | NCL-DYS2  NCL-DYS3 | 1:300  1:100 |
| **F4/80** | Abcam | ab6640 | 1:200 |
| **CD206** | Abcam | ab8918 | 1:50 |
