# Supplementary figures and images for: Human dental pulp pluripotent-like stem cells promote wound healing and muscle regeneration
Source: Stem Cell Res Ther. 2017 Jul 27;8:175. doi: 10.1186/s13287-017-0621-3 (PMC5531092; doi:10.1186/s13287-017-0621-3)

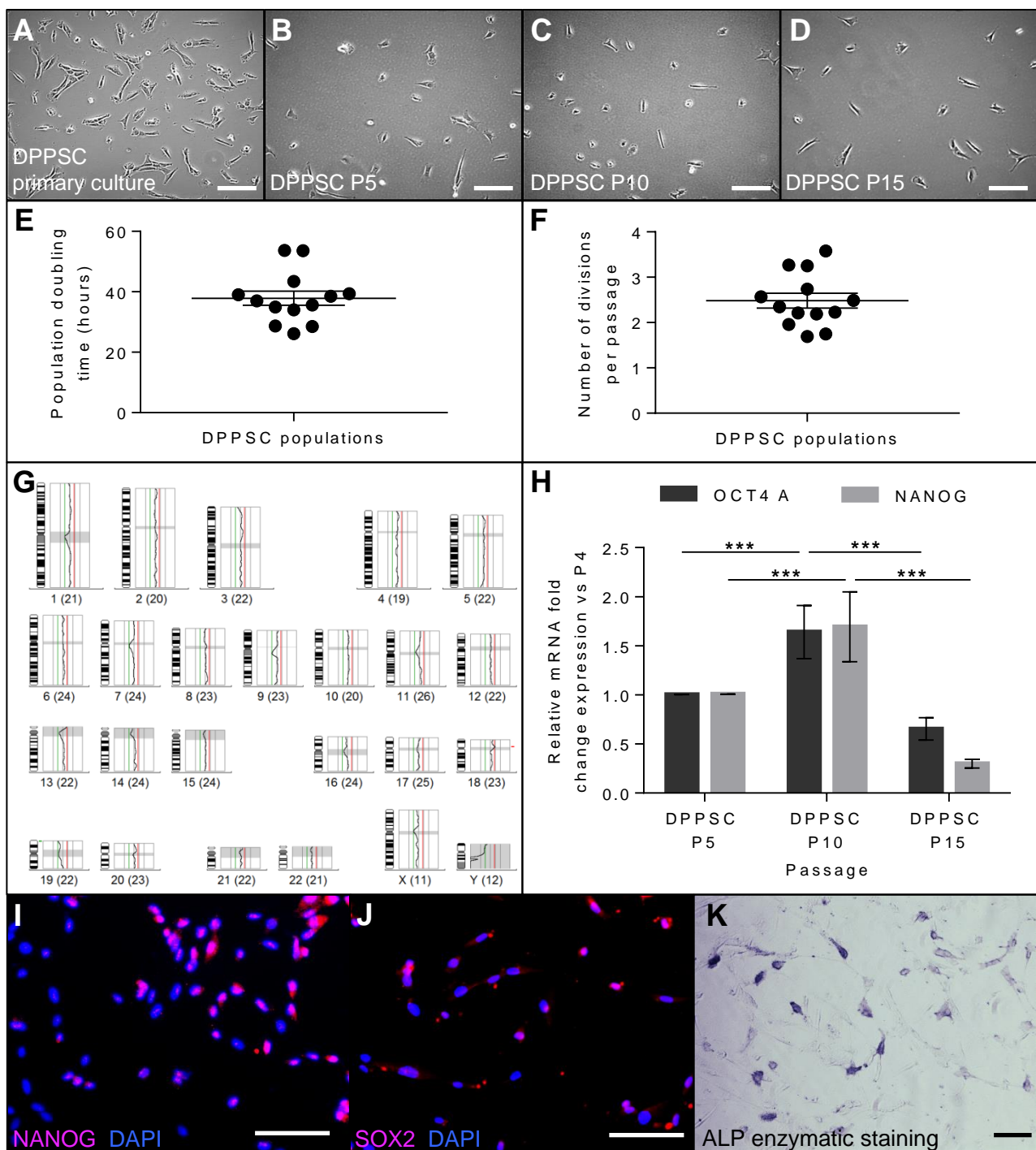

Supplement: Supplementary file 3 — DPPSC characterisation. a-d Phase contrast images of DPPSC morphology in the primary culture (a), passage (P)5 (b), P10 (c) and P15 (d). Scale bars: 200 μm. e Population-doubling time in hours of DPPSC for 15 passages. n = 13 different donors, results are displayed as mean ± s.e.m.. f Number of divisions per passage of DPPSCs for 15 passages. n = 13 different donors, results are displayed as mean ± s.e.m. g Example of a short-Comparative Genomic Hybridisation in DPPSC at P15 showing no chromosomal abnormalities. The DNA control used for the hybridisation was XXY, therefore the observed loss of chromosome Y indicates these cells are from a female donor. h Relative mRNA fold change expression of the pluripotency markers OCT4A and NANOG at P5, P10 and P15 compared to P5 in DPPSC from eight different donors. ***p < 0.001, n = 8 different donors, two-way ANOVA was used and results are displayed as mean ± s.e.m. i, j Immunofluorescence analyses for the pluripotency markers NANOG (red; i) and SOX2 (red; j) in undifferentiated DPPSC at P10. Nuclei are counterstained with DAPI (blue). Merge is observed in purple. Scale bars: 100 μm. k Alkaline phosphatase (ALP) staining of DPPSC at P10. Scale bar: 100 μm. (PDF 196 kb) [file 13287_2017_621_MOESM3_ESM.pdf]

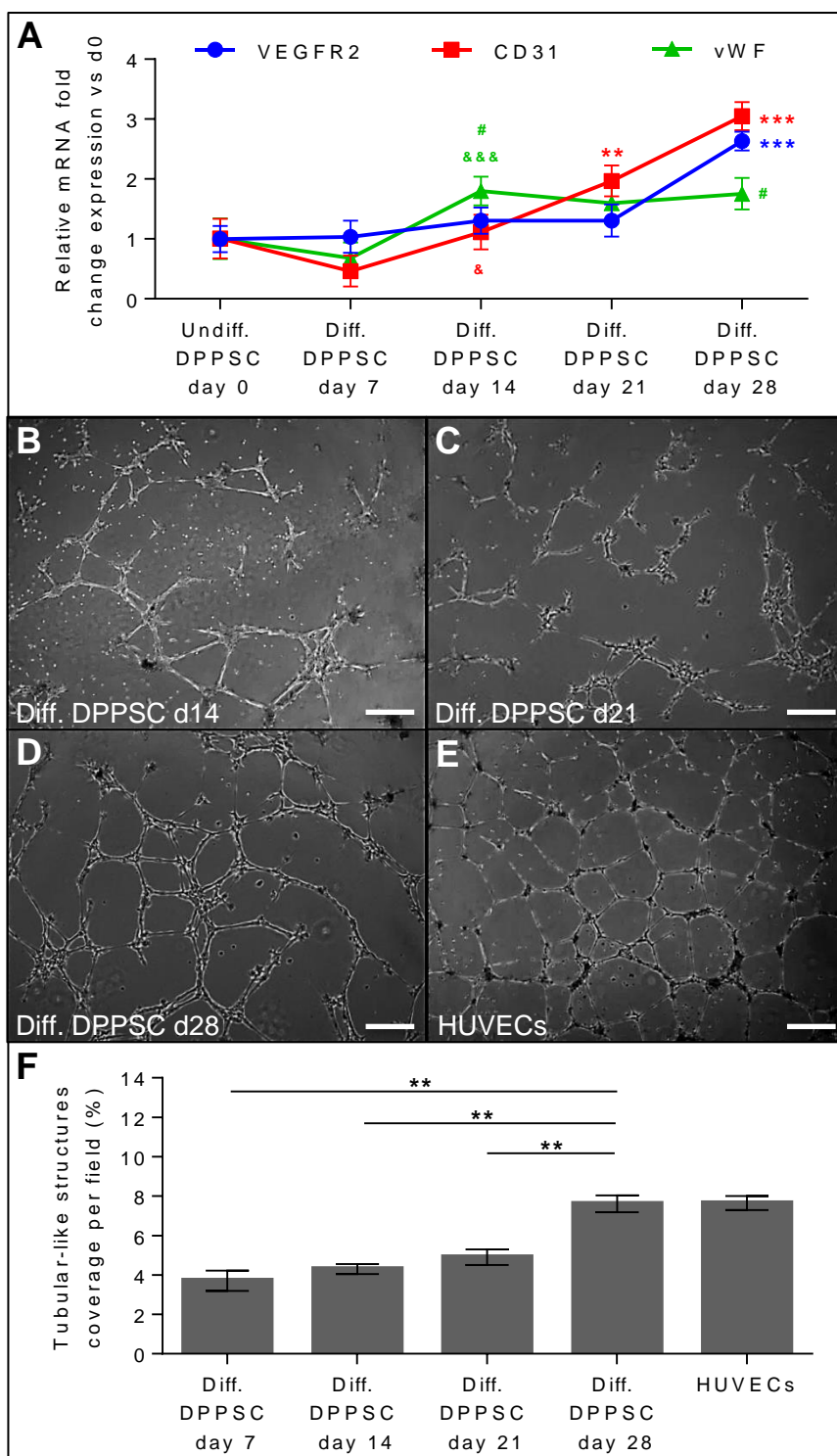

Supplement: Supplementary file 4 — In vitro endothelial differentiation of DPPSC. a qRT-PCR of the endothelial markers VEGFR2, CD31 and vWF at different differentiation time points. HUVECs were used as controls and GAPDH as housekeeping gene. *p < 0.05, **p < 0.01, ***p < 0.001, # p < 0.05 (vWF gene expression d0 vs d14; d0 vs d28), &p < 0.05 (CD31 gene expression d7 vs d14), &&&p < 0.001 (vWF gene expression d7 vs d14), n = 3 independent experiments, one-way ANOVA was used, results are displayed as mean ± s.e.m.. b-e Functional 2D Matrigel™ assay at 24 hours showing tube-like structures formed by DPPSC at day 14 (b), 21 (c) and 28 (d) of endothelial differentiation and by HUVECs (e). Scale bars: 500 μm. f Quantitative analysis of the tubular-like structures formed in the Matrigel™ assay by HUVECs and differentiated DPPSC at different time points. Data obtained by differentiated DPPSC at day 28 are statistically significant compared to those obtained in the other time points. **p < 0.01, n = 3 independent experiments, one-way ANOVA was used, results are displayed as mean ± s.e.m. (PDF 113 kb) [file 13287_2017_621_MOESM4_ESM.pdf]

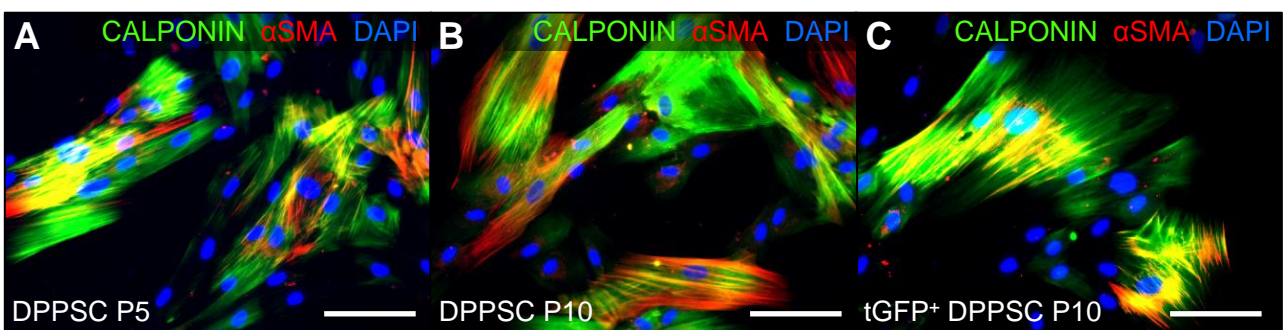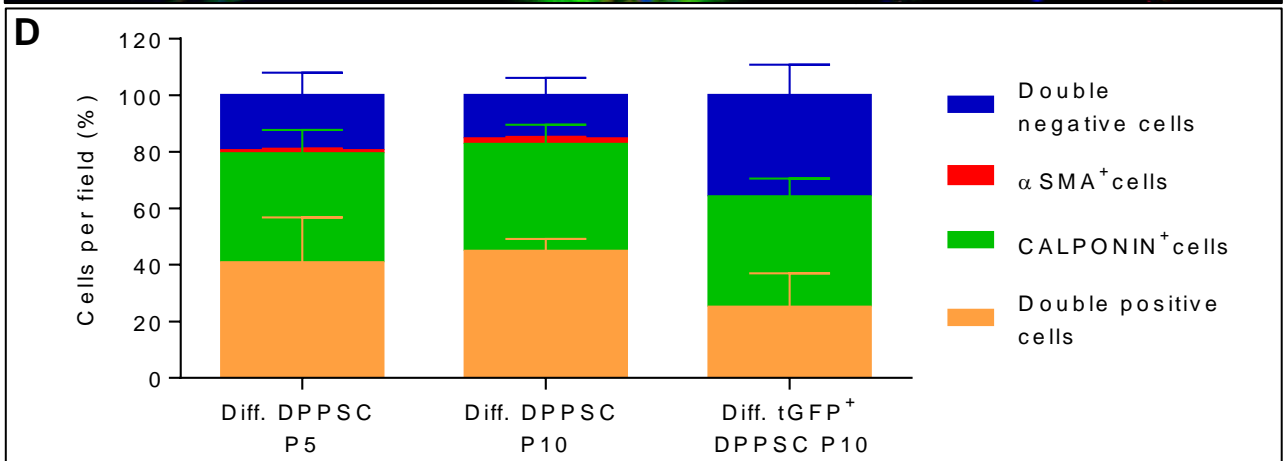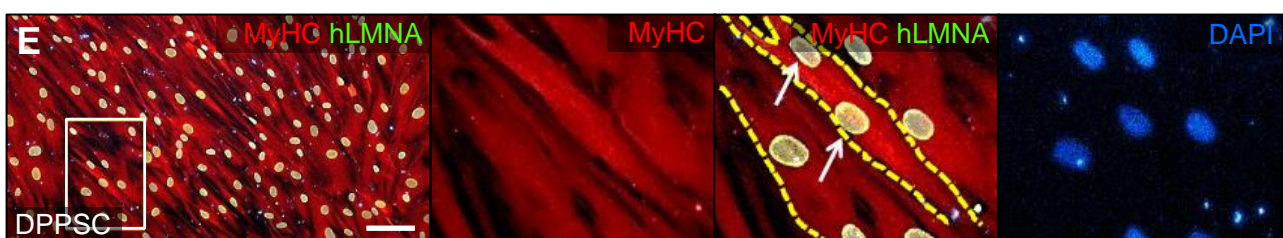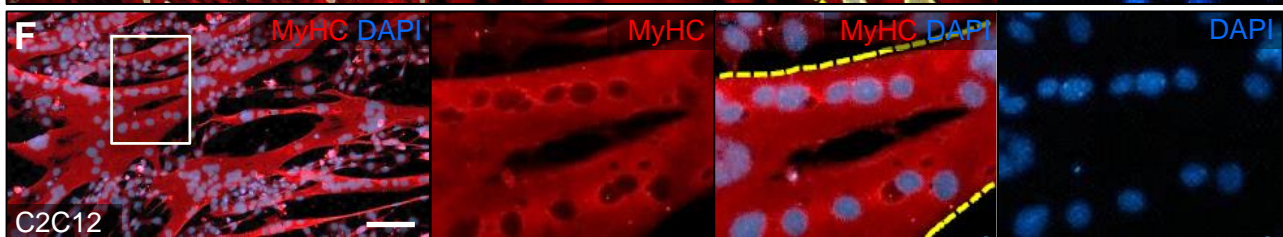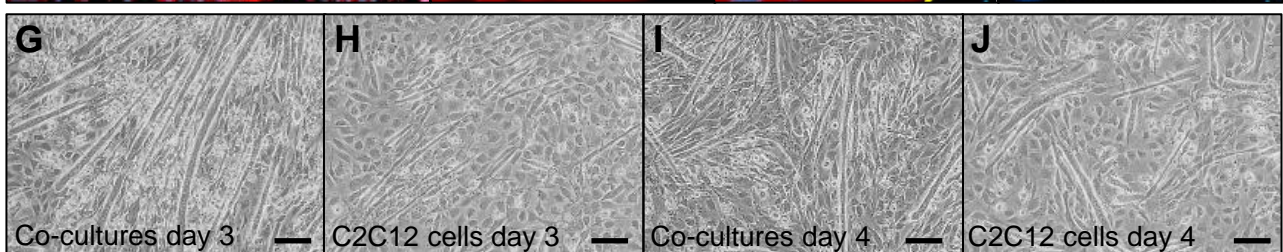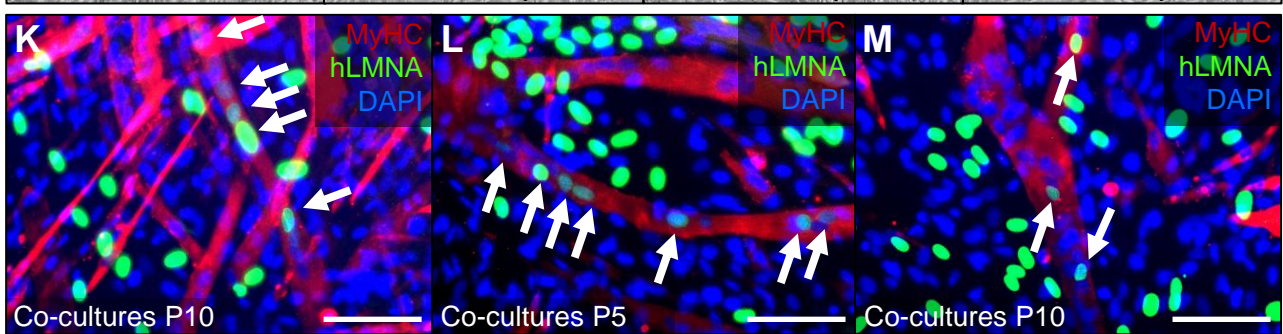

Supplement: Supplementary file 5 — In vitro smooth muscle and myogenic differentiation of DPPSC. a-c Immunofluorescence analysis for the smooth muscle markers αSMA (red) and calponin (green) in DPPSC from a different donor than Fig. 2d, e cultured in differentiation medium for 10 days at P5 (a) and P10 (b). tGFP+ DPPSC from the same donor at P10 were also analysed (c). Double-positive cells are shown in orange. Nuclei are counterstained with DAPI (blue). Scale bars: 100 μm. d Quantitative analysis of the percentage of cells expressing both smooth muscle markers (orange), calponin alone (green), αSMA alone (red) or none of these markers (blue), showing no statistically significant difference. n = 3 independent experiments, one-way ANOVA was used, results are displayed as mean ± s.e.m.. e Immunofluorescence of DPPSC differentiated for 7 days to skeletal muscle. MyHC is shown in red and hLMNA in green. Yellow lines show the presence of myotubes with more than one nucleus inside. Nuclei are counterstained with DAPI (blue). f Immunofluorescence of the mouse myoblast cell line C2C12 differentiated for 7 days to skeletal muscle. MyHC is shown in red. An example of a myotube is also indicated with yellow lines. Nuclei are counterstained with DAPI (blue). g-j Co-cultures of DPPSC-C2C12 (g, i) or C2C12 mono-cultures (h, j) at day 3 (g, h) or 4 (i, j) of skeletal muscle differentiation. k DPPSC from the same donor as Fig. 2g at P10 co-cultured with C2C12 cells for 5 days. Arrows indicate the presence of human nuclei (stained for hLMNA in green) inside the formed myotubes expressing MyHC (red). Nuclei are counterstained with DAPI (blue). l, m DPPSC from another donor at P5 (l) and P10 (m) co-cultured with C2C12 cells for 5 days. Arrows indicate the presence of human nuclei (stained for hLMNA in green) inside the formed myotubes expressing MyHC (red). Nuclei are counterstained with DAPI (blue). For e-m, scale bars: 100 μm. (PDF 344 kb) [file 13287_2017_621_MOESM5_ESM.pdf]

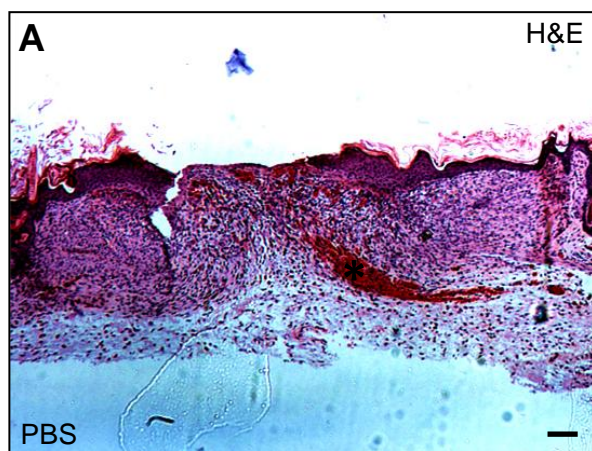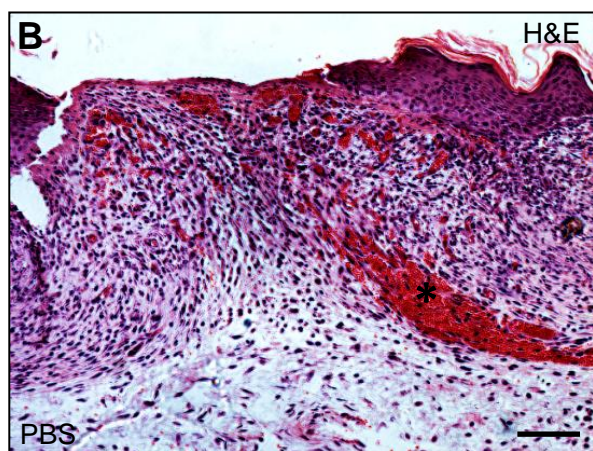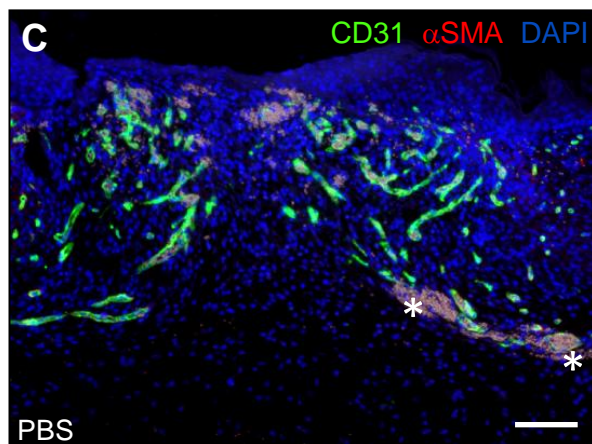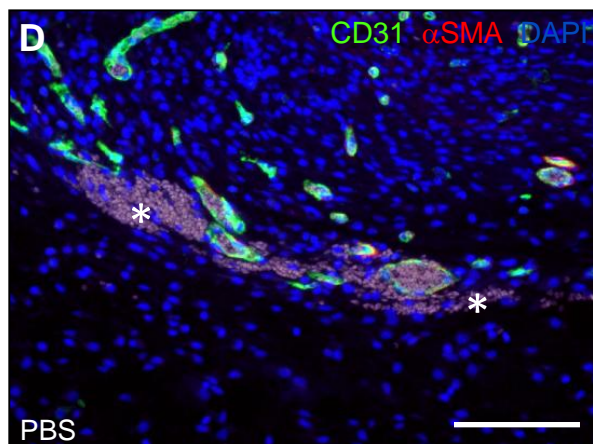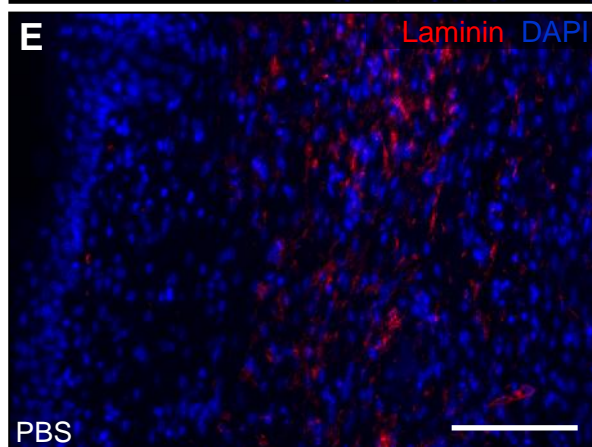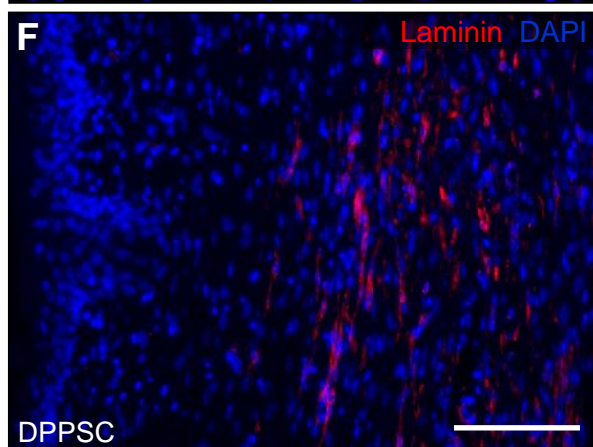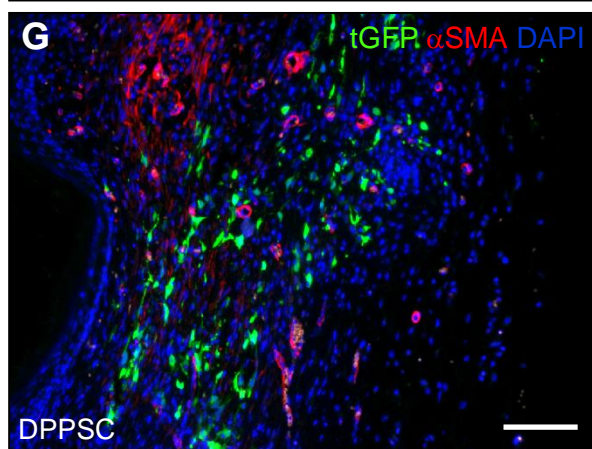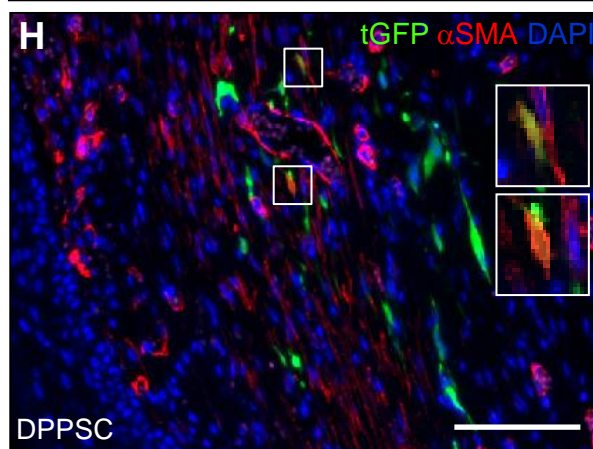

Supplement: Supplementary file 6 — Effect of DPPSC on leakage and dermal healing. a, b Haematoxylin and eosin staining of paraffin sections of the wound tissue in PBS-treated wounds. Presence of red blood cell (RBC) leakage (indicated by asterisks) can be observed. Scale bars: 100 μm. c, d CD31 (green) and αSMA (red) immunofluorescence analysis in serial sections of a, b, showing the presence of autofluorescent RBC outside of the CD31+ vessels in PBS-treated wounds. RBC leakage is indicated by asterisks. e, f Laminin (red) immunofluorescence analysis in PBS (e) or DPPSC-treated (f) wounds for the analysis of the basement membrane of the endothelium. g, h tGFP (green) and αSMA (red) double staining on wound cross-sections of DPPSC-treated mice revealed very few double positive cells (yellow; h) not associated with vessel-like structures. For c-h, nuclei are counterstained with DAPI (blue); scale bars: 100 μm. (PDF 496 kb) [file 13287_2017_621_MOESM6_ESM.pdf]

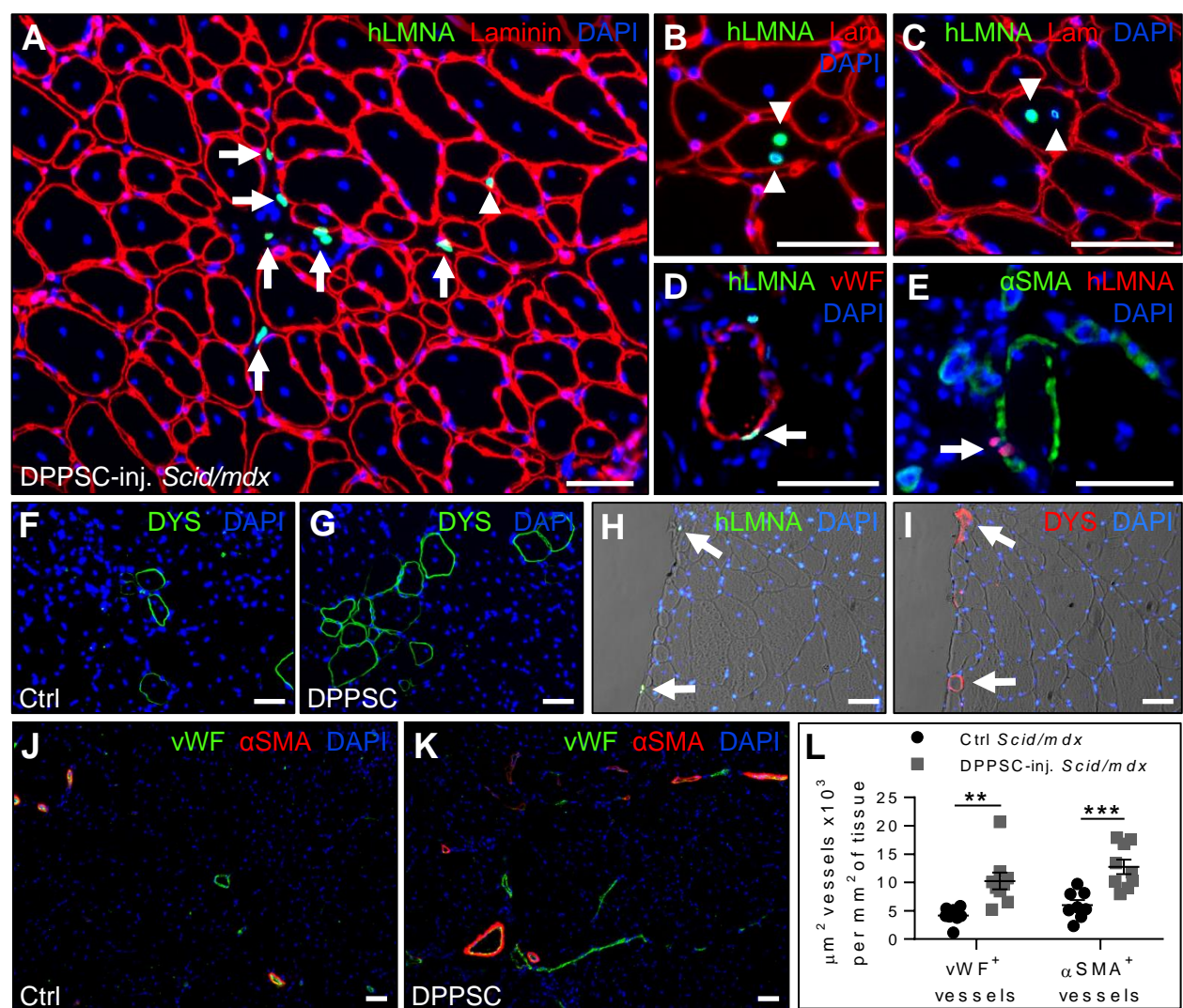

Supplement: Supplementary file 7 — DPPSC engraftment, differentiation and revascularisation in Scid/mdx dystrophic mice at day 20. a-c Immunofluorescence analysis for hLMNA (green) and laminin (red), showing DPPSC engraftment in Scid/mdx mice. Arrows indicate DPPSC in the interstitial space, while arrowheads indicate localisation in the basal lamina or integration inside the fibres. d Immunofluorescence analysis for hLMNA (green) and vWF (red) in DPPSC-injected Scid/mdx. e Immunofluorescence analysis for hLMNA (red) and αSMA (green) in DPPSC-injected Scid/mdx. f, g Immunofluorescence analysis for dystrophin (DYS; in green) in control (f) and DPPSC-injected Scid/mdx (g) muscles. h, i Immunofluorescence analyses in two serial sections for hLMNA (green; h) and dystrophin (red; i) in DPPSC-injected Scid/mdx mice. Bright field allows the identification of the same fibres in the two serial sections. j, k vWF (green) and αSMA (red) immunofluorescence analysis in control (j) or DPPSC-injected (k) Scid/mdx muscles. For a-k, nuclei are counterstained with DAPI (blue); scale bars: 50 μm. l Quantitative analysis of the area of vWF+ or αSMA+ vessels per mm2 of tissue in Scid/mdx muscles. **p < 0.01, ***p < 0.001, n = 9 mice/group. Two-tailed Student’s t test was used and results are displayed as mean ± s.e.m.. (PDF 281 kb) [file 13287_2017_621_MOESM7_ESM.pdf]

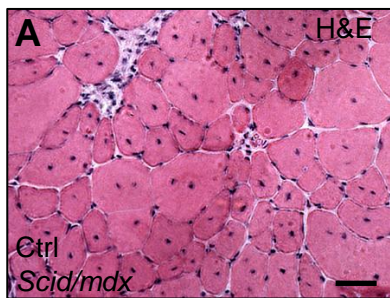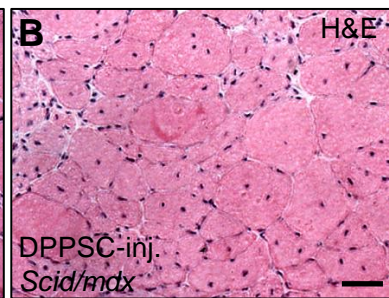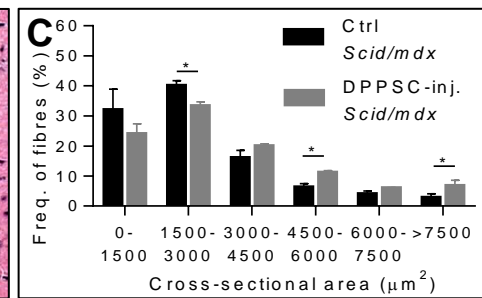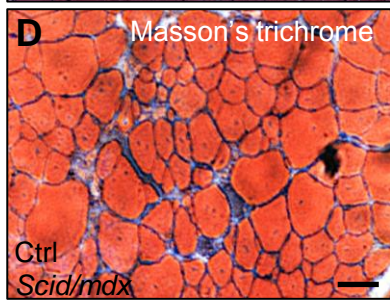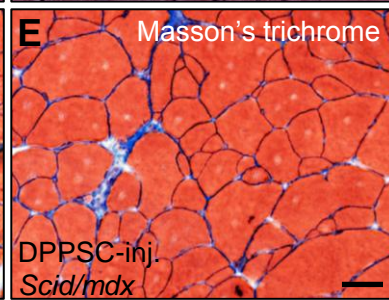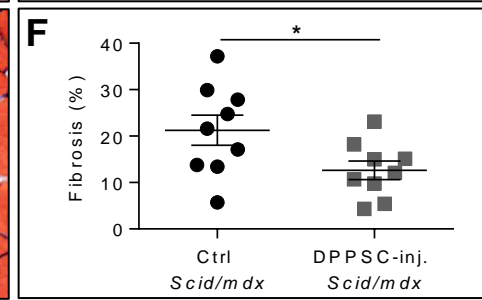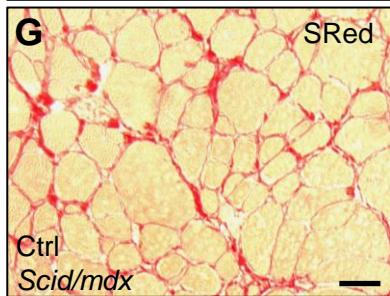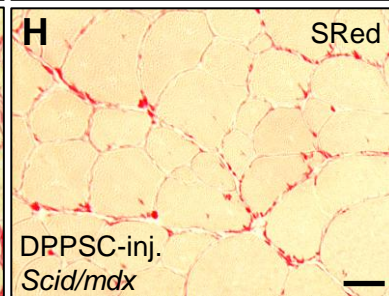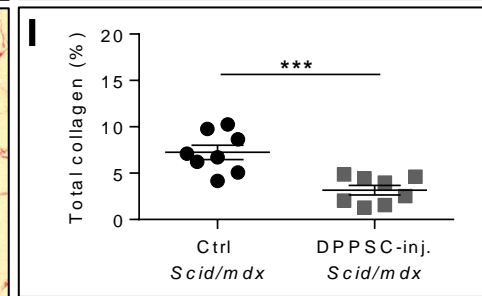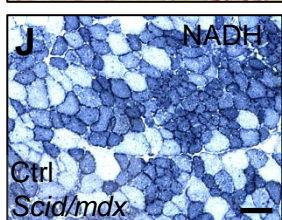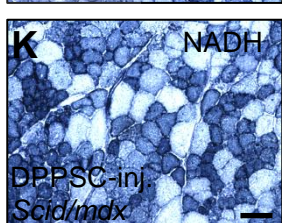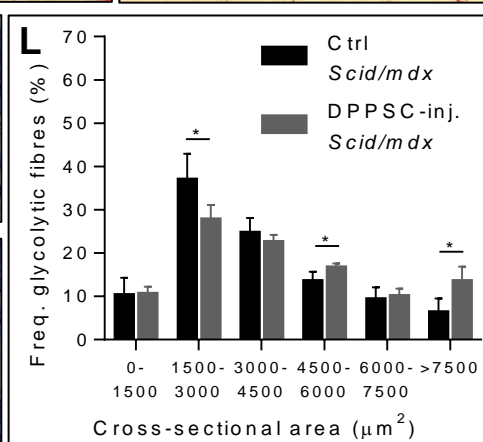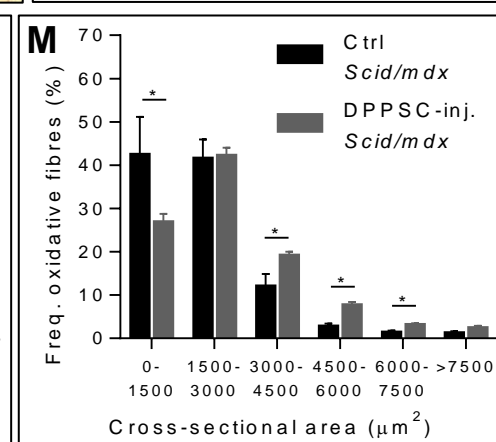

Supplement: Supplementary file 8 — Histological, morphometric and fibre type analyses after DPPSC injection in dystrophic Scid/mdx mice. a, b Haematoxylin and eosin staining in control (a) or DPPSC-injected (b) Scid/mdx muscles. Scale bars: 100 μm. c Quantitative frequency distribution analysis of the cross-sectional area of the fibres in Scid/mdx muscles. *p < 0.05, n = 3 for each group. d, e Masson’s trichrome staining in control (d) or DPPSC-injected (e) Scid/mdx muscles revealing areas of fibrosis (blue). Scale bars: 100 μm. f Quantitative analysis of the percentage of fibrosis per field in Scid/mdx muscles. *p < 0.05, n = 9 for each group. g, h Sirius Red staining visualised by bright field microscopy in control (g) or DPPSC-injected (h) Scid/mdx muscles for the analysis of total collagen. Scale bars: 100 μm. i Quantification of the total collagen present in Scid/mdx muscles. ***p < 0.001, n = 8 for each group. j, k NADH staining of control (j) and DPPSC-injected (k) Scid/mdx muscles, showing oxidative fibres in blue and glycolytic fibres in white. Scale bars: 100 μm. l, m Quantitative frequency distribution analysis of the cross-sectional area of type II fast-glycolytic (l) or type I slow-oxidative (m) fibres in the NADH staining in Scid/mdx muscles injected with DPPSC compared to control muscles. *p < 0.05, n = 3 for each group. For c, f, i, l, m, two-tailed Student’s t test was used and results are displayed as mean ± s.e.m.. (PDF 310 kb) [file 13287_2017_621_MOESM8_ESM.pdf]

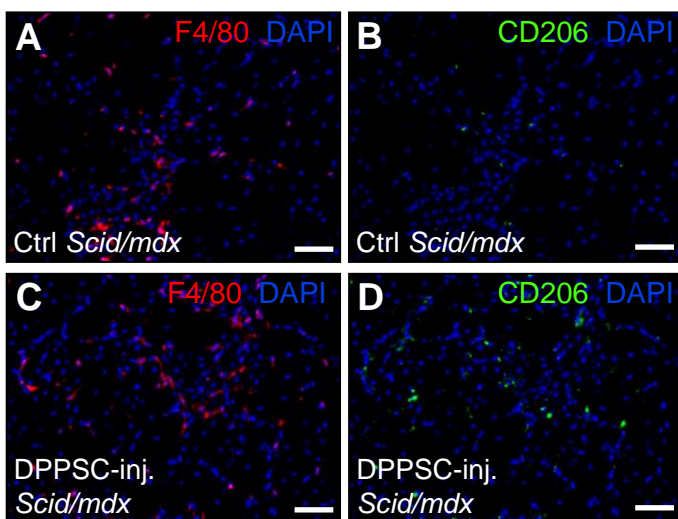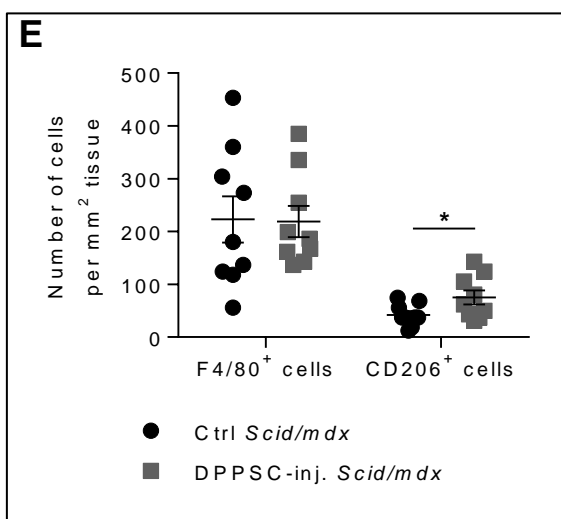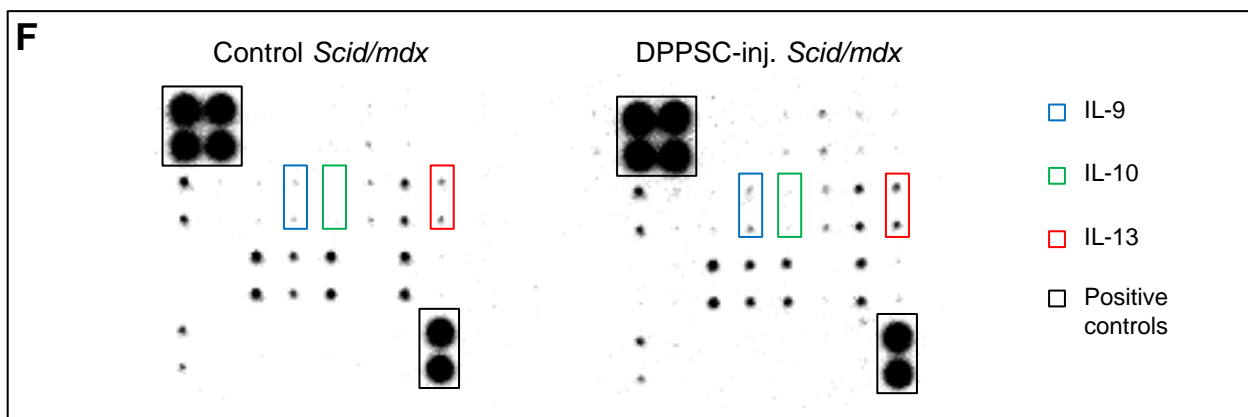

Supplement: Supplementary file 9 — Macrophage and cytokine analyses after DPPSC injection in dystrophic Scid/mdx mice. a-d Immunofluorescence analysis of macrophage-specific F4/80 (red; a, c) and proangiogenic M2 macrophage-specific CD206 (green; b, d) in control (a, b) or DPPSC-injected (c, d) Scid/mdx muscles. Scale bars: 50 μm. e Quantitative analysis of the number of cells expressing F4/80 and CD206 macrophage markers per mm2 of tissue in Scid/mdx muscles. *p < 0.05, n = 9 for each group; two-tailed Student’s t test was used and results are displayed as mean ± s.e.m.. f Cytokine antibody arrays showing the apparent increment in spot intensity in IL-9, IL-10 and IL-13 in DPPSC-injected (right panel) compared to control (left panel) Scid/mdx muscles. (PDF 51 kb) [file 13287_2017_621_MOESM9_ESM.pdf]
